# Is dental amalgam safe for humans? The opinion of the scientific committee of the European Commission

**DOI:** 10.1186/1745-6673-6-2

**Published:** 2011-01-13

**Authors:** Joachim Mutter

**Affiliations:** 1 Department of Environmental and integrative medicine Lohnerhofstraße 2, 78467 Constance/Germany

## Abstract

It was claimed by the Scientific Committee on Emerging and Newly Identified Health Risks (SCENIHR)) in a report to the EU-Commission that "....no risks of adverse systemic effects exist and the current use of dental amalgam does not pose a risk of systemic disease..." [1, available from: http://ec.europa.eu/health/ph_risk/committees/04_scenihr/docs/scenihr_o_016.pdf].

SCENIHR disregarded the toxicology of mercury and did not include most important scientific studies in their review. But the real scientific data show that:

(a) Dental amalgam is by far the main source of human total mercury body burden. This is proven by autopsy studies which found 2-12 times more mercury in body tissues of individuals with dental amalgam. Autopsy studies are the most valuable and most important studies for examining the amalgam-caused mercury body burden.

(b) These autopsy studies have shown consistently that many individuals with amalgam have toxic levels of mercury in their brains or kidneys.

(c) There is no correlation between mercury levels in blood or urine, and the levels in body tissues or the severity of clinical symptoms. SCENIHR only relied on levels in urine or blood.

(d) The half-life of mercury in the brain can last from several years to decades, thus mercury accumulates over time of amalgam exposure in body tissues to toxic levels. However, SCENIHR state that the half-life of mercury in the body is only "20-90 days".

(e) Mercury vapor is about ten times more toxic than lead on human neurons and with synergistic toxicity to other metals.

(f) Most studies cited by SCENIHR which conclude that amalgam fillings are safe have severe methodical flaws.

## Dental amalgam is the main source of mercury in human tissues

SCENIHR (Scientific Committee on Emerging and Newly Identified Health Risks) from the European Commission claim [1]: "Exposure to mercury is difficult to measure. The indications for mercury exposure are therefore normally obtained by measuring mercury levels in urine and blood of individuals."

SCENIHR did not cite any autopsy studies, which are the most reliable studies for assessing mercury levels in tissues.

An approx. 2-5-fold increase of mercury levels in blood and urine in living individuals with dental amalgam as well as a 2-12 fold increase in several body tissues was observed in deceased individuals with dental amalgam [2-21]. Additionally, studies with animals have confirmed the fact that dental amalgam leads to significantly increased levels in the tissues [22-28].

According to these studies, dental amalgam is responsible for at least 60-95% of mercury deposits in human tissues. This was not acknowledged by SCENIHR.

## No organic mercury compounds through dental amalgam?

SCENIHR [1] state that "there is no evidence that biotransformation of amalgam derived mercury takes place intra-orally in association with bacterial activity."

In contrast to this claim studies have shown that mercury (Hg) from dental amalgam is transformed into organic mercury compounds by microorganisms in the human gastrointestinal tract [29-31]. Leistevuo et al. (2001) found a three-fold increase of methylmercury levels in saliva of individuals with dental amalgam compared to individuals without amalgam, although frequency and kind of fish consumption were identical in both groups. Mercury levels in saliva exceed mercury limits for sewage in 20% of individuals with amalgam [30]. The form of methylmercury derived from dental amalgam may be much more toxic (up to 20 times) than the form of methylmercury found in fish (see section "toxicity of mercury").

## Toxic mercury levels in vitro and in vivo

Inorganic mercury levels of 0.02 ng Hg/g (2 μl of 0.1 μMolar Hg in 2 ml substrate) led to the total destruction of intracellular mircrotubuli and to the degeneration of axons [32]. In other experiments inorganic mercury levels of 36 ng Hg/g (0.18 μMol Hg) led to increased oxidative stress as a prerequisite for further cell damage [33,34].

Mercury vapor inhalation in doses which also occur in humans with many amalgam fillings and chewing led to pathological changes in the brains of animals after 14 days [35,36].

## No toxic mercury levels in humans through dental amalgam?

In a recent autopsy study, it was found that individuals with more than 12 amalgam fillings have more than 10-times higher mercury levels in several tissues including the brain, compared to individuals with only 0-3 amalgam fillings [11].

The average mercury level in the brain of EU citizens with more than 12 amalgam fillings was 300 ng Hg/g brain tissue [11], which is well above mercury levels proven to be toxic in vitro on neurons (0.02 -36 ng Hg/g) (see above).

In another autopsy, individuals with more than 10 amalgams have 504 ng Hg/g in their kidney tissues (0-2 amalgams: 54 ng Hg/g) and 83.3 ng Hg/g in the liver (0-2 amalgams: 17.68 ng Hg/g) [5].

Mercury levels in thyroid- and pituitary glands were 55 ng Hg/g and 200 ng Hg/g respectively and again, these levels correlates significantly with numbers of amalgam fillings [37].

Because the levels found in these studies are only average levels, a significant portion of individuals with dental amalgam have more than twice (standard deviation) these toxic mercury levels in their body tissues. It is important to note that mercury levels found in subcellular fractions like microsomes, mitochondria and other cell compartments even exceed the average levels of the brain samples analysed in these studies [38].

## Toxic mercury levels in Alzheimer's disease

The average mercury load in brain tissues of individuals with Alzheimer`s disease was 20 to 178 ng Hg/g; in some cases the load exceeded up to (236- 698 ng Hg/g). In 15% of the human brain samples the mercury load was above 100 ng Hg/g [39-41]. The average mercury load in the pituitary gland was 400 ng Hg/g [42]. These levels are again well far above established toxic levels (see above).

## Pathological changes, caused by mercury, in most german human brains?

About 20% of individuals in the age group of 20 years, 50% of individuals in the age group of 50 years, and 90% of people in the age group of 85 years living in Germany show pathological changes in their brains that are typical for Alzheimer's disease [43] and mercury toxicity. This coverage of pathological brain changes caused by very low levels of mercury in experiments and not by low levels of other metals (like lead, iron, aluminum, copper, manganese, chromium, cadmium) [32,36] resembles the frequency of dental amalgam fillings implanted in humans: About 80-90% of people living in Germany have dental amalgam over decades. It must be noted that about 30-50% of german people above the age of 85 years have Alzheimer's disease (AD) and there are many hints that mercury plays the major pathogenetic role in AD [44].

## Maternal amalgam as the main source of mercury in infant tissues

Maternal amalgam fillings lead to a significant increase of mercury levels in fetal and infant body tissues including the brain [6]. Furthermore, placental, fetal and infant mercury body burden correlates with the number of amalgam fillings of the mothers [6,45-52].

Mercury levels in amniotic fluid [53] and breast milk [54-56] also significantly correlate with the number of maternal amalgam fillings.

## Mercury in infant tissues: Increased risk of neurodevelopmental disorders?

Drasch et al. found mercury levels of up to 20 ng Hg/g in German infant brain tissues which were mainly caused by dental amalgam fillings of their mothers [6]. As described above, mercury levels of 0,02 ng Hg/g led to degeneration of axons [32]. Furthermore, the mercury levels found in the brains of infants whose mothers were dental amalgam bearers are sufficient enough to inhibit the function of the important enzyme methionin synthetase [57,58]. Methionin synthetase is crucial for methylation, a central step for most important metabolic reactions the the body, including the development of the brain, the maturation of nerve cells and the production of neurotransmitters.

Maternal amalgam fillings also increase significantly mercury levels in cord blood [59,60]. The risk for delayed neurodevelopment of children was 3.58- times increased when mercury levels in cord blood were higher than 0.8 ng Hg/ml [61]. It is important to note that mercury levels of 0.2 - 5 ng Hg/ml cord blood are considered "normal" in Germany [62], thus leaving many infants with mercury levels that may cause neurodevelopmental deficits.

## No correlation between mercury in urine or blood and in body tissues

The SCENIHR report is based on studies which have measured mercury levels in biomarkers such as urine for the assessment of clinical symptoms or mercury body burden. However, the WHO states (1991) that

"Mercury typifies a "retention" toxicity and much of the mercury taken into the body is absorbed by the solid tissues. The amount in urine represents mercury being excreted. However, the main question is how much is being retained in the different body tissues".

It has been shown in experiments with animals and men that in spite of normal or low mercury levels in blood, hair and urine high mercury levels are found in critical tissues such as brain and kidney [7,13,20,22,25,28,46,63,64]. A recent study on deceased individuals confirm that there exists no correlation between inorganic mercury levels in urine or blood and mercury levels in brain tissues [37].

Drasch and coauthors have shown that 64% of individuals occupationally exposed to mercury vapor and having typical clinical signs of mercury intoxication had urine levels of mercury below 5 μg/l, which represent the No Observed Adverse Effect Level (NOAEL). The same results were found for mercury levels in blood and hair [65-67].

## Paradoxical association between mercury levels in urine and clinical symptoms

There is even a paradoxical correlation between mercury levels in urine, blood or hair and clinical symptoms: Subjects with highest urine levels of mercury showed best recovery rates from neuropsychological complaints after removing their amalgam fillings [68]. Also children with highest mercury levels in hair showed better performance in developmental tests [69]. Another study indicates that in spite of a significantly higher exposure to mercury in their mothers` womb autistic children had up to 15-times lower mercury levels in their infant hair than healthy children [46]. Furthermore, the lower the mercury levels in infant hair, the higher was the severity of autism [46].

Despite higher mercury body burden, a "amalgam hypersensitivity" group showed slightly lower levels of mercury in their saliva, blood and urine [70]. Even after provocation with the mercury chelator DMPS, the "amalgam hypersensitivity" group excreted in mean only 7,77 μg Hg via urine in 24 h whereas healthy amalgam bearers excreted 12,69 μg Hg/24h [70].

Furthermore, studies confirm that the ratio of fecal to urine excretion is 12 to 1 [13]. This proves that the majority of excreted mercury leaves through the bilary transport system of the liver via the fecal route. Urine mercury therefore represents a minor excretory route of less than 8% of mercury being excreted. Also, urine mercury is a measure of mercury being excreted by the kidney---not a measure of total mercury body burden.

## Safety levels for mercury?

In view of the data presented above, it is impossible to determine any safety levels below which adverse effects can be excluded [71]. SCENIHR used safety limits which were deduced from studies with workers occupationally exposed to mercury. However, these limits cannot be applied to individuals with amalgam fillings and must be critically evaluated:

a) Frequently, mercury exposure of workers in the chlorine-alkali industry is used for comparison although the simultaneous exposure to chlorine considerably diminishes the absorption of mercury into the body tissues of animals by 50-100% [72].

b) Workers exposed to mercury usually represent a group whose mercury-exposure starts only with adulthood (for about 8 hours a day, 5 days a week), while amalgam bearers can be exposed to mercury in the womb through maternal amalgam fillings during their childhood and until death at a rate of 24 hours per day, 7 days per week.

c) Workers are a selected healthy group, while pregnant women, infants, children and individuals with illnesses (such as multiple sclerosis, autoimmunity, cancer, psychiatric diseases) do not start working at all either due to industrial safety regulations or to early health problems during working.

d) Despite mercury exposure below "safety limits", significant adverse health effects were found in most studies in workers exposed occupationally to mercury, even several years after the exposure had ceased [73-81].

## Body half-time period of mercury

SCENIHR state that the body half-time (of mercury) is "20-90 days".

Particularly in the brain, mercury has a significantly longer half-time of more than 17 years [63,64,82-87].

## Toxicity of mercury

SCENIHR did not mention the specific toxicity of mercury vapor coming off dental amalgam fillings. This should be mentioned in a risk analysis:

Mercury has been shown to be 10 times more toxic than lead (Pb) in vitro [88-90]. Mercury is the most toxic non-radioactive element. Mercury vapor is one of the most toxic forms of mercury along with some of the organic mercury compounds. This extraordinary toxicity is also determined by the following properties:

a) Mercury is the only metal representing a volatile gas at room temperature, which is readily absorbed (80%) by the respiratory system.

b) Mercury vapor from amalgam penetrate into tissues with great ease, because of its monopolar atomic configuration.

c) Once inside the cells, mercury vapor is oxidized to Hg^2+^, the very toxic form of mercury which binds covalently to thiol groups of proteins inhibiting their biological activity.

d) Hg^2+ ^is more toxic than Pb^2+^, Cadmium (Cd^2+^) and other metals because it has a higher affinity due to "covalent bond" formation with thiol groups (cysteines in proteins) causing irreversible inhibition. Other metals form reversible bonds with proteins and are therefore less toxic.

e) Hg^2+ ^does not bind tightly enough to the carboxylate groups of natural organic acids (natural chelators like citrate) for its toxicity to be prevented.

f) Chelating agents, like EDTA, which normally inhibit the toxic effect of heavy metals like lead, have no inhibitory effect on the toxicity of mercury or may even increase it [91,92]. Other chelating agents (DMPS and DMSA) inhibit the toxic effect of Cd^2+ ^and Pb^2+, ^but not of Hg^2+ ^[93]. DMPS, DMSA or natural chelators like vitamin C, glutathione or alpha-lipoic acid are not able to remove mercury from nervous tissues [94]. DMPS or DMSA may even increase the inhibitory activity of Hg^2+ ^and Cd^2+^on enzymes but not of Pb^2+ ^[95]. Furthermore, DMPS in animals led to an increase of mercury concentrations in the spinal cord [96].

The toxicity of methylmercury which is bound to cysteine in fish seems to be far lower (only approx. 1/20) than methylmercury compounds usually used in experiments [97].

Furthermore, marine fish represents a significant source of selenium and essential omega-3-fatty acids, which are known to protect effectively against mercury toxicity. Nevertheless, methylmercurychloride, which proved to be more toxic than methylmercury in fish, showed less neurotoxicity for the growing nervous system in vivo than did mercury vapor [98].

Investigations by Drasch et al. show similar correlations: The population of a goldmining area, which was exposed to mercury vapor, showed significantly more neurological symptoms of mercury intoxication than a control group which mainly was exposed to methylmercury from fish consumption, despite their mercury levels in hair and plasma being higher compared to the individuals exposed to mercury vapor [65,66]. Another study also points to smaller neurotoxicity of methymercury from fish compared to iatrogenic mercury sources (amalgam, thimerosal) [46]. Here, in contrast to the numbers of dental amalgam in the mothers, no correlation between maternal fish consumption during pregnancy and the risk of autism for their children was found.

In summary, mercury vapor coming off dental amalgam or methylmercury derived from amalgam in the gastrointestinal tract has not reacted with anything yet and has the full toxic potential. On the other hand, methylmercury in fish has already reacted with fish proteins and other protective molecules or atoms in fish tissues such as glutathione or selenium, which are enriched in fish. Furthermore, newest studies confirm that most individuals with dental amalgam fillings are exposed to toxic mercury levels [99,100].

## Synergistic toxicity of mercury to lead (Pb)

Some scientists try to argue that results gained by animal or cell testing are overestimated and not comparable to the situation of the human body. However, in contrast to test animals in experiments, humans are exposed to many other toxins simultaneously, thus the effects add up or are even synergistic [101,102]. For example, it has been proven that the combination of the Lethal Dose 1% of mercury (LD1_Hg_) together with the LD1 of lead (Pb) results in the death of all animals, so the following toxicological equation can be assumed: LD1 (Hg) + LD1 (Pb) = LD 100 [101].

In this context, it must be considered that modern humans have more mercury and between 10-1,000-times more lead in their body tissues than ancient humans.

In other experiments, the addition of aluminumhydroxide (often in vaccines), antibiotics, thimerosal (sometimes in vaccines) and testosterone increased the toxicity of mercury [108,109]. The synergistic toxicity of testosterone explain the observation, that much more males than females suffers from autism or amyotrophic lateral sclerosis.

## No adverse effects through dental amalgam?

SCENIHR states "It is generally concluded that no increased risk on adverse systemic effects exists and we do not consider that the current use of dental amalgam poses a risk of systemic disease" and "....some local adverse effects are occasionally seen with dental amalgam fillings, but the incidence is low and normally readily managed"

SCENIHR has neglected numerous scientific studies which find significant adverse health effects from dental amalgam:

## Cytotoxicity of amalgam in comparison to composites

SCENIHR compare the toxicity of amalgam with composites. However, in most experiments, even inorganic mercury, which is much less toxic than mercury vapor (because inorganic mercury is not able to penetrate easily into the cells), was proven to be much more toxic than any composite compound: Mercury was shown to be 100-800- fold more toxic than composite components for human cells [110-114].

## Genotoxicity, oxidative stress, cancer

Dental amalgam fillings have been found to cause DNA damage in human blood cells. [115]. Even low levels of inorganic mercury lead to significant DNA damage in human tissue cells and lymphocytes [116]. This effect, which trigger cancer, has been found with mercury levels below those normally causing cytotoxicity and cell death. Furthermore, aberrations of chromosomes can be provoked by amalgam in cell cultures [117]. Amalgam bearers show significantly increased oxidative stress in saliva [118,119] and blood [120,121]. The increase of oxidative stress correlates with the numbers of amalgam fillings. Mercury levels normally seen in tissues of individuals with amalgam fillings lead to increased oxidative stress and reduction of glutathione levels, thus inducing cellular damage [33,34]. Significantly elevated mercury levels have also been observed in breast cancer tissues [122]. Mercury deposited in the tissue is mostly bound to selenium, which means that the this selenium is no longer available for the body. Therefore, amalgam may aggravate a latent deficiency of selenium, particularly in countries with suboptimal selenium supply (e.g. in Central Europe) [123,124].

## Antibiotic resistance

It has been shown that mercury from dental amalgam can induce mercury resistant bacteria [125-127]. This leads to a general antibiotic resistance in oral bacteria and in other body sites [127], which is particularly true when the antibiotic resistance genes are contained within the same mobile element as the mercury resistance operon [128,129]. Mercury resistance is common in human oral bacteria [130,131]. Monkeys with dental amalgam also showed an increase in antibiotic resistant bacteria in their stools [127,132].

## Penetration of amalgam in tooth bone and jaw

Experiments on monkeys and sheep have shown that mercury from amalgam penetrates easily into the dentin roots as well as into the jaw bone [25,26]. The fact that this was also shown for humans [133] confirms an alternative route of mercury exposure caused by amalgam.

## Skin

There is a correlation between atopic eczema and IgE-levels and the body burden of mercury [134]. Amalgam fillings can induce lichenoid reactions [135-139]. In more than 90% of the cases, these lesions have been found to recover upon removal of amalgam, no matter whether an allergy patch test was positive or not. Granulomatosis improved likewise [140]. Also, other forms of dermatitis seem to be related to dental amalgams [141,142].

## Autoimmune disorders and mercury hypersensitivity

Constant low-dose mercury exposure, as is common in amalgam bearers, has been considered a possible cause for certain autoimmune diseases, e.g. multiple sclerosis, rheumatoid arthritis or systemic lupus erythematosus (SLE) [135,143-152]. These effects occur with exposure below mercury safety limits [153]. Recent research has shown that mercury and ethylmercury have the ability to inhibit the first step (phagocytosis) in the innate and acquired immune response of humans at very low levels [154]. This shows that mercury exposures quite below the average exposure through amalgam exposure can cause disruption of the immune system at all ages.

## Only "rare cases of proven allergic reactions"?

SCENIHR only accept the "proof" of allergic reactions to amalgam, which is a positive cutaneous patch test. However, it has been shown that in more than 90% of the cases with mucosal reactions these lesions have been found to recover by removal of amalgam, no matter whether a cutaneous patch test was positive or not [137,139,140]. Therefore the relevance of the cutaneous patch test in detecting sensitivity or allergy to mercury implanted in the oral cavity without any epicutaneous contact has been severely questioned [155].

The results with another validated test system reveal that there are more than just "rare cases" with immunological complaints due to dental amalgam [148,150,152,156-162].

There may also be a correlation between atopic eczema, IgE-levels and the body burden of mercury, which is also not detected by means of cutaneous patch tests [134].

Because mercury from maternal dental amalgam is one of the main sources of mercury body burden in fetal and infant tissues, postnatal atopic eczema disappear after mercury detoxification of the infants [163].

## Heart diseases

Mercury may cause hypertension and myocardial infarction [164].

Significant mercury accumulation (22,000 times higher than controls) has been found in heart tissues with a form of heart insufficiency [165].

## Urinary system

SCENIHR cited only one review performed by a dentist and published in a dental trade journal [166] as well as 5-7 year studies on initially healthy children, also performed mainly by dentists, to back up their argument that "there is no evidence that dental amalgam fillings affect kidney function in humans". However, there are many other studies suggesting quite the opposite:

In animal experiments, an impairment of renal functions due to amalgam fillings has been observed [23,146,167]. Humans with amalgam fillings show more signs of tubular and glomerular damage when compared to individuals without dental amalgams [15]. The frequently mentioned children amalgam trial study found first signs of kidney damage (microalbuminuria) [168] even after only 5 years of amalgam exposure.

## Alzheimer's disease (AD)

SCENIHR questioned that mercury may contribute to the development of AD. As a proof of this statement they cited only one study [41] published in the trade journal of the world-wide leading American Dental Association (ADA) [102]. In contrast, other studies have shown that mercury play a major pathogenetic role in AD [108,109,169,170]. A new systematic analysis of the literature regarding the role of mercury in AD found a significant association [124].

## Parkinson's disease (PD)

Heavy metals have long been suspected to be a cause of PD, with several studies showing a relation, including epidemiological studies [171-180]. Elemental mercury has induced PD [175], and in a case report, the condition of PD substantially improved after treatment with a mercury chelator [173] and remained unchanged during a 5-year follow-up period [173]. In another study, significantly elevated blood mercury levels were found in 13 of 14 patients with PD compared to healthy controls [172]. This supports the conclusion of a previous study which found a correlation between blood mercury levels and PD [176]. Another study found significantly higher amalgam exposure in individuals with PD compared to healthy controls [179].

## Adverse health effects in dental staff?

SCENIHR state that "the incidence of reported adverse effects [in dental staff and dentists] is very low".

A simple literature research reveals quite the opposite: Dentists working with amalgam have an increased mercury exposure [17,181,182]. In most studies available, mercury exposure in dental clinics resulted in significant adverse health effects in dental workers. In some studies, the clinical outcome was not correlated with mercury levels in urine or blood, and some authors falsely concluded that mercury was therefore not the cause of the adverse effects. However, this is not scientific since urine- or blood mercury levels did not correlate with tissue levels (see above). Lindbohm et al. (2007) found a two-fold increased risk for miscarriage through occupational exposure to mercury (OR 2,0; 95% CI 1,0- 4,1). The effect from mercury exposure was stronger than from exposure to acrylate compounds, disinfectants or organic solvents [199].

Even 30 years after cessation of mercury exposure, dental nurses showed significant adverse health effects [200]. In spite of the fact that 85% of the dentists and dental technicians tested showed mercury related toxicities in both behavior and physiological parameters, and 15% showed increased mercury induced neurological deficits with polymorphism of the CPOX4 gene [186,188,201], SCENIHR still maintain that amalgams do not cause any significant medical problems in dental workers, because urine and blood levels are below "safety limits".

## Infertility

SCENIHR stated that "There is no evidence of any association between amalgam restorations and either male or female fertility or obstetric parameters". As a proof of this statement, SCENIHR cited just one study, which examines only semen parameters in men. However, other studies point to the opposite direction, especially when examining women:

Female dental assistants exposed to amalgam showed a higher rate of infertility [198]. Women with more amalgam fillings or increased mercury levels in urine (after mobilization with DMPS) had a higher incidence of infertility [202-204]. Heavy metal detoxification led to spontaneous pregnancies in a considerable part of the infertile patients [203]. Exposure to mercury also lead to decreased male fertility [205-207]. A Norwegian study which is often cited as a proof that mercury exposure in dental clinics does not cause infertility suffers from methodological flaws insofar as only women were included who had already given birth to at least one child. Women without children were excluded. Such a study certainly cannot answer the question if working with amalgam leads to infertility or not. Moreover, the exposure time to amalgam was not calculated and thus not included as a covariate into the study.

## Multiple Sclerosis (MS)

A 7,5-fold increased level of mercury was found in the cerebrospinal fluid (CSF) of MS patients [208]. It would be difficult to speculate that the presence of this increase in the CSF would not at least exacerbate the problems associated with MS or any other neurological disease. The prevalence of MS has been shown to be correlated with the prevalence of caries [209,210] and the prevalence of amalgam [211,212]. Several MS epidemics occurred after acute exposure to mercury vapor or lead [213]. In animal models inorganic mercury caused a loss of Schwann cells which build the myelin sheaths and stabilize the axons of neurons [214]. Autoimmune pathogenesis, including antibodies against myelin basic protein (MBP), can be provoked by mercury and by other heavy metals [148].

MS patients who had their amalgam fillings removed showed fewer depressions, less aggression and less psychotic and compulsory behaviors when compared to a group of MS patients with amalgam fillings [215]. They also had significantly lower levels of mercury in blood [216]. After amalgam removal, pathological oligoclonal bands in the CSF disappeared in MS patients [217]. Removal of dental amalgam also led to a recovery in a significant proportion of MS patients [147]. A retrospective study on 20,000 military individuals revealed a significantly higher risk for MS in individuals with more amalgam fillings [218]. This risk was underestimated, because the study cohort which was selected by means of medical examination consisted exclusively of individuals with good health at the time of joining the military [218]. Another problem occurring in some studies is the absence of documentation of the dental status before or at the time of the onset of multiple sclerosis. In spite of these limitations [219] a reanalysis found a 3.9- fold increased risk for multiple sclerosis in individuals with amalgam compared to individuals with no amalgam. A recent systematic review also found an increased risk for MS caused by dental amalgam in spite of the fact that most studies did not use proper amalgam-free controls [220].

## Amyotrophic Lateral Sclerosis (ALS)

SCENIHR state that "there is no evidence for a relationship between Amyotrophic Lateral Sclerosis (ALS) and mercury"

In contrast to the statement of SCENIHR, there are many studies which suggest that mercury may play a pathogenetic role in ALS:

Mercury vapor is absorbed by motor neurons [221] where it leads to increased oxidative stress. In experiments, mercury vapor was found to promote motor neuron diseases such as ALS [222-226]. It was proofed that mercury enhances glutamate toxicity in neurons, which is one factor in ALS. Case reports show a correlation between accidental mercury exposure and ALS [227,228]. There is a reported case of a Swedish woman with more than 34 amalgam fillings who suffered from ALS. After removal of these fillings she recovered [229]. A retrospective study reported a statistically significant association between an increased number of amalgam fillings and the risk of motor neuron diseases [218].

## "Amalgam disease" and markers of sensitivity

Among the most frequently reported symptoms due to amalgam fillings are: Chronic fatigue, headache, migraine, increased susceptibility to infections, muscle pain, lack of concentration, digestion disorders, sleeping disorders, low memory capacity, joint pain, depression, heart sensations, vegetative disregulation, mood disorders and many more [161,215,216,230-234].

Until recently it was not possible to differentiate between „amalgam-sensitive" and „amalgam-resistant" persons by their mercury levels in blood or urine or an epicutaneous test (patch test) [9,21]. However, it could be shown that subjects could react to a mercury patch test with psychosomatic complaints, although there was no allergic reaction of the skin [235]. In addition, neutrophil granulocytes in amalgam-sensitive subjects react differently compared to those in amalgam-resistant subjects [236] and different activities of the superoxide dismutase could be found [237].

## Increased susceptibility to mercury and amalgam

SCENIHR did not mentioned any susceptibility parameters which make a significant proportion of the population more susceptible to mercury from dental amalgam:

### a) Abnormal porphyrine profiles due to mercury exposure

It is known that mercury exposure leads to aberrant urine porphyrine profiles in dentists [238] and autistic children and that this aberrancy was reversed by treating these children with a mercury chelator [239-241].

A genetic polymorphism of coproporphyrinoxidase (CPOX4) [188,201] leads to increased susceptibility to mercury and thus to a higher risk for neurobehavioral complaints [242].

The critical question here is the effect of mercury vapor exposure on brain porphyrine profiles since an aberrancy in brain heme has been associated with the inability to remove beta-amyloid protein from brain cells, which in turn may lead to Alzheimer's disease [243].

It should be noted that porphyrins lead to heme, and heme is critical for several biochemical mechanisms: (i) heme is the oxygen carrying cofactor for haemoglobin, (ii) heme is a critical cofactor for the P450 class of enzymes that are responsible for detoxifying xenobiotics from the body, (iii) heme is a necessary cofactor for one of the complexes in the electron transport system of mitochondria and therefore ATP-synthesis.

Therefore, mercury inhibition of heme production could have a multitude of secondary effects causing human complaints and illnesses.

In spite of the fact that 85% of the dentists and dental technicians tested showed mercury related toxicities in both behavior and physiological parameters, and 15% showed an increase of mercury induced neurological deficits with polymorphism of the CPOX4 gene, organized dentistry and SCENIHR still maintain that amalgams do not cause any significant medical problems because the urine and blood levels are below safety limits.

### b) Brain derived neurotrophic factor

Another genetic polymorphism of the brain derived neurotrophic factor (BNDF) increases also the susceptibility to very low level mercury exposure [186,187].

### c) Apolipoprotein E diversity

It could be shown that amalgam sensitive persons are significantly more likely to be carriers of the apolipoprotein E4-allel (APO-E4) than symptom free controls and that they are less likely to carry the APO-E2 [231,234]. APO-E4 is known to be the major genetic risk factor for Alzheimer's disease, whereas APO-E2 decreases the risk. It has been postulated that this is due to the difference in capacity to remove heavy metals from the cerebrospinal fluid [44,92,102,124,231,234,244]. APO-E2 possesses two cysteines with metal binding sulfhydryl-groups whereas APO-E4 does not have any cysteine residues.

### d) Glutathione metabolism

Reduced glutathione (GSH) is the main natural chelator for heavy metals in the body due to its sulfhydryl-containing cysteine. Only mercury, which is bound to glutathione (or selenium), is capable of leaving the body via urine or biliary excretion. Thus, a high level of glutathione is crucial for mercury metabolism. It has been described that polymorphisms in genes leading to impaired GSH production cause higher retention of inorganic and organic mercury in the body. Other factors which may increase susceptibility to low dose mercury exposure, e.g. low levels of selenium, abnormal reaction of neutrophil granulocytes, activity of super oxide dismutase, D4-receptor positive methionine synthetase and impaired methionine transulfuration- and methylation pathways (about 15% of the population), led to decreased mercury protecting agents, like S-adenyl-methionine, cysteine, GSH and metallothionine [44,245-247].

## Improvement after removal of amalgam

Significant improvement of health and above mentioned diseases (including Multiple Sclerosis and other autoimmune diseases) have been reported after amalgam removal (in most studies with elaborate protective measures to minimize mercury exposure) [68,147,149,150,159,161,217,230,233,234,248-251].

## No neurodevelopmental disorders through mercury?

SCENIHR stated that "There is no evidence of a causal relationship between dental amalgam and autism" and "... that no link has been yet established between vaccines, thimerosal and autism".

Nonetheless other authors come to opposite conclusions:

"...mercury exposure altered cell number and cell division; these impacts have been postulated as modes of action for the observed adverse effects in neuronal development. The potential implications of such observations are evident when evaluated in context with research showing that altered cell proliferation and focal neuropathologic effects have been linked with specific neurobehavioral deficits (e.g., autism)." [252]

Cheuk and Wong (2006) in patients diagnosed with attention-deficit hyperactivity disorder and Desoto and Hitlan (2007) in patients diagnosed with autistic disorders found significant elevations in blood mercury levels in comparison with controls [253,254]. Adams et al. (2007) observed significant increases in the mercury levels of baby teeth in infants with autistic disorders in comparison with controls [255]. Mercury in baby teeth mirrors mercury exposure in the womb.

Recent brain pathology studies have revealed elevations in mercury levels and mercury-associated oxidative stress markers in patients diagnosed with autistic disorders. The level of mercury in the urine of autistic children shows an increase of 3-5 times after appropriate treatment with the mercury chelator DMSA compared to healthy children [259]. Autistic children also excrete higher concentrations of coproporphyrine which is specific for mercury intoxication [239,240,260,261]. Detoxification of mercury with DMSA normalizes the abnormal coproporphyrin levels in autistic children [239,240] and led to improvement of symptoms [262]. Additionally, experimental as well as epidemiological studies indicate that mercury exposure is responsible for autism or a deterioration of the disease. Prenatal exposure to maternal amalgam [46,263], maternal thimerosal [46,264] and postnatal sources (mercury from vaccines for the child) together with a genetic sensitivity may trigger autism. In animal experiments vaccination with thimerosal led to symptoms similar to autism [265]. Epidemiological studies confirm a significant association between low-dose mercury exposure and neurodevelopmental disorders [266][267][268][269][270][271]. Autistic children show decreased levels of the natural mercury chelator glutathione [272]; it is known that mercury is capable of causing this phenomen [273]. In some preliminary therapy studies with chelation therapy led to improvement of symptoms [263]. The Autism Research Institute therefore lists chelation as the most effective therapeutic approach among 88 therapies including 53 medications [274].

Zahir et al. (2005) described that the access of mercury

"...to man through multiple pathways air, water, food, cosmetic products and even vaccines increase the exposure. Fetuses and infants are more susceptible to mercury toxicity. Mothers consuming diet containing mercury pass the toxicant to fetuses and to infants through breast milk. Decreased performance in the areas of motor function and memory has been reported among children exposed to presumably safe mercury levels [...] Mercury has been found to be a causative agent of various sorts of disorders, including neurological, nephrological, immunological, cardiac, motor, reproductive and even genetic. Recently heavy metal mediated toxicity has been linked to diseases like Alzheimer's, Parkinson's, Autism, Lupus, Amyotrophic lateral sclerosis, etc."[275].

Some studies which found no associations between mercury exposure and autism have severe methodical flaws [245].

## Severe methodical flaws in studies cited by SCENIHR as a proof of the safety of dental amalgam

In order to study toxic effects it is necessary to compare at least two samples: one that was exposed to the substance in question and one that was not. One of the main problems in most of the amalgam studies is that the vast majority did not incorporate a true control group that had never been exposed to dental amalgam. Even when comparing samples with and without dental fillings, the sample without dental fillings had been exposed to dental amalgam earlier in life. The studies cited frequently not only by SCENIHR as a proof of the putative harmlessness of amalgam do not use "proper" non-amalgam control groups. There is a prominent example to describe:

The Swedish twin study [276] actually only compared 57 twin-pairs in a co-twin analysis, and not 587 as mentioned by the authors and many governmental institutions. As the average age of the sample was 66 years, 25% had no teeth at the time of investigation, many had missing teeth and an unknown number had crowns using other dental materials. Root fillings with amalgam and amalgam fillings under crowns were not calculated. As an allegedly "non-amalgam" group, they were compared with individuals who still had teeth with amalgam fillings. The authors found that individuals with more amalgam fillings (which means also more own teeth) had a better health status. It is fair to assume that individuals with few or no teeth or teeth that have been restored with crowns or bridges had probably had dental amalgam previously. As mercury accumulates in body tissues, this "amalgam free group" might have a higher mercury body burden than the "amalgam group" with currently existing amalgam fillings.

SCENIHR also cited Zimmer et al. (2002) as a proof of the safety of amalgam. But this study compared two groups exposed to amalgam (all female, one group of patients who claimed to be suffering from symptoms they related to their amalgam fillings and the other group which did not report any association between complaints and their amalgam) in terms of mercury levels in body fluids and psychometric tests. The mean number of amalgam fillings was identical in both groups. They found equal mercury levels in both amalgam groups. Zimmer et al. (p. 210) conclude: "Thus, mercury released from amalgam fillings was not a likely cause of complaints reported by the amalgam sensitive subjects" [21]. It is not clear why these authors come to such a conclusion. Furthermore it is known from animal experiments and pharmacological studies that individuals given equal amounts of a toxin might react differently. An example for this is that not every smoker develops lung cancer, although smoking is now accepted as a main cause for cancer.

## "Children amalgam trials"

SCENIHR based their statement about the safety of dental amalgam also on two children amalgam trials. These studies show severe methodical flaws:

In two randomised trials on children it was evaluated whether mercury containing dental amalgam had adverse neuropsychological or renal effects [277,278]. Healthy children were randomised to either amalgam or composite surface restoration. Two children in the amalgam group died (one possibly by committing suicide) and were excluded from the study.

Power calculation (binomial - adverse event versus no event) indicates that psychological illness, having prevalence of 6.7% in the composite-treated children, would have to have had a prevalence of at least 14.5% in the amalgam group to have an 80% chance of being proven statistically (observed was 9.0%). Similarly for neurological illness, observed prevalences in the composite group (0.4% composite, 1.5% amalgam) would have needed at least 4.5% prevalence in the amalgam group to be significant. From the authors it was concluded that "there is no reason to discontinue use of mercury amalgam" [277] and that "dental amalgam [...] emits small amounts of mercury vapor" [278].

The first conclusion is a classic error: Due to its lack of power, the study provides false reassurance that mercury is 'safe'. To effectively evaluate the effect sizes seen, the trial should have been much larger (1500-2500/group).

Urine porphyrin profiles and markers of oxidative stress, which are elevated in individuals with dental amalgam [19,119] were not measured. Also, genetic polymorphism, which increase the susceptibility to mercury, like BDNF-Polymorphism [186,188] and Glutathion-S-Transferase gene polymorphism [279] were not measured either. Furthermore, the real exposure level of mercury (mercury vapor emitted in the oral cavity) was not determined, which questions the ethics of such a study. Research has demonstrated that the emission of mercury vapor was much higher than what has been "estimated" by dentists. Chew et al. (1991) showed that 43.5 microgram/cm^2^/day mercury was released from a "non-mercury releasing amalgam" and this remained constant over the study period of 2 years [280].

Mean mercury urine levels were significantly higher in the amalgam groups [277,278], although in years 3 to 7 the levels of mercury in the urine of the amalgam bearers continuously dropped until they approached the levels of the amalgam free children [278]. But restorative treatment was used in years 6 and 7, which should have increased or at least maintained the urine mercury levels. This needed explaining. In the Chew study above [280], the amount of mercury released was steady for 2 years (the length of the study). It is known that amalgam do not stop releasing mercury vapor within 7 years. The question therefore is what the drop was caused by after year 2? Urine mercury levels are a measure of the amount of mercury being excreted via this route. Therefore, after two years of mercury exposure the route of kidney excretion of mercury appears to be becoming less effective. This is consistent with the well-known fact that increased mercury exposure inhibits its own excretion. It has been published and verified that over 90% of mercury excreted by humans leaves through the biliary transport system of the liver and is excreted in the feces, not in the urine [13]. The conclusion of Bellinger et al. [277] that "there is no reason to discontinue use of mercury amalgam" is amazing, because possible adverse effects may need more than five years of mercury exposure to develop. If mercury is involved in the pathogenesis of Alzheimer's disease, the disease may need up to 50 years to be diagnosed clinically [44].

One of the included criteria for the two studies was "no interfering health conditions" including neurodevelopmental disorders. The Centers for Disease Control and Prevention (CDC) in Atlanta (USA) reports that 1 in 6 American children have a neurodevelopmental disorder. However, above mentined papers conclude that amalgams should remain a viable clinical option in dental restorative treatment [278] and they did not exclude use on children with neurodevelopmental disorders - exactly the type of child, however, which they excluded from their studies. As mercury exposure during pregnancy may be the prime cause of neurodevelopmental disorders [46,61,245], this conclusion from the children amalgam is unsafe for the public.

## Amalgam for mercury pollution

There has been an alarmingly increase of mercury in our environment [281] and human bodies [282] over the last decades. The UNEP reports on a 3-5 fold increase over the last 25 years [281].

In the European Union (EU) the usage of amalgam amounts to 120 tons yearly. Dentists are the 2^nd ^largest user group in the EU [283,284].

Recent calculations done by Hylander [284,285] show that there are 40 tons of mercury in teeth with dental amalgam of Swedish people, which results to the excretion of 100 kg of mercury per year in wastewater. 1300 to 2200 tons of mercury in dental amalgam is present in the teeth of citizens in the EU (27 countries) [284], and for the USA the respective figures are about 1000 tons. In the US, dental amalgam is the 3rd most significant source of environmental mercury [286]. In contrast to the EU, removed amalgam is not separated from the wastewater of dental clinics in the US. But even in most EU-countries, where such separators are in use, parts of the dental amalgam leaks into the environment [284].

This mercury from dental amalgam (i.e. mercury emissions from dental clinics in wastewater, excreted mercury emissions from amalgam in living individuals, mercury emissions from elevated mercury deposits in tissues of deceased and cremated humans with dental amalgam) enter into the environment. When including environmental costs into the economic calculation (except costs from amalgam related diseases), amalgam is the most costly dental material as was shown by Hylander and Godsite [283].

## The role of organized dentistry in SCENIHR and in defending amalgam

The SCENIHR amalgam expert group consisted of one engineer (chairman), four dentists, a toxicologist and two veterinarians. The chairman has tight contacts to the industry. No experts for medicine or environmental medicine were included. One must wonder why it were the dentists who represented the strongest party in SCENIHR.

Due to their education and clinical experience, dentist are not able to judge medical systemic adverse side effects caused by dental amalgam, like multiple sclerosis, autism, autoimmunity, Alzheimer's disease, psychiatric diseases etc. Usage of dental amalgam may increase worldwide (increasing caries epidemic in undeveloped countries which constitute the highest percentage of the world`s population ). Today, dental organisations are the only trade group of health professionals who endorse the use of a product that is primarily made of mercury. Every amalgam patent has been produced according to dental organisations specifications [287,288]. This may indeed be a critical point, because organized dentistry, which has always support the use of dental amalgam, are responsible for adverse side effects [287,288]. Therefore, the strategies of organized dentistry used to influence science and politics over the last decades [287-290] may be analogous to other well known topics with existing conflicts of interest, where effective measures have been applied to influence science and politics regarding dangerous products [291-295].

## Competing interests

The author declare that he have no competing interests.

## References

[B1] Scientific Committee on Emerging and Newly Identified Health Risks (SCENIHR) The safety of dental amalgam and alternative dental restoration materials for patients and users Europaen Commision 2008 1 74 http://ec.europa.eu/health/ph_risk/committees/04_scenihr/docs/scenihr_o_016.pdf

[B2] Barregard J Svalander C Schutz A Westberg G Sällsten G Blohmé I Mölne J Attman PO Haglind P Cadmium, mercury, and lead in kidney cortex of the general Swedish population: a study of biopsies from living kidney donors Environ Health Perspect 1999 107 867 871 10544153 10.1289/ehp.107-1566723 PMC1566723

[B3] Becker K Kaus S Krause C Lepom P Schulz C Seiwert M Seifert B German Environmental Survey 1998 (GerES lll): environmental pollutants in blood of the German population Int J Hyg Environ Health 2002 205 297 308 10.1078/1438-4639-00155 12068749

[B4] Becker K Schulz C Kaus S Seiwert M Seifert B German Environmental Survey 1998 (GerES III): Environmental pollutants in the urine of the German population Int J Hyg Environ Health 2003 206 15 24 10.1078/1438-4639-00188 12621899

[B5] Drasch G Schupp I Riedl G Günther G Einfluß von Amalgamfüllungen auf die Quecksilberkonzentration in menschlichen Organen Dtsch Zahnärztl Z 1992 47 490 496

[B6] Drasch G Schupp I Hofl H Reinke R Roider G Mercury burden of human fetal and infant tissues Eur J Ped 1994 153 607 610 10.1007/BF02190671 7957411

[B7] Drasch G Wanghofer E Roider G Are blood, urine, hair, and muscle valid bio-monitoring parameters for the internal burden of men with the heavy metals mercury, lead and cadmium? Trace Elem Electrolyt 1997 14 116 123

[B8] Eggleston DW Nylander M Correlation of dental amalgam with mercury in brain tissue J Prosth Dent 1987 58 704 707 10.1016/0022-3913(87)90424-0 3480359

[B9] Gottwald B Traencker I Kupfer J Ganss C Eis D Schill WB Gieler U "Amalgam disease" -- poisoning, allergy, or psychic disorder? Int J Hyg Environ Health 2001 204 223 229 10.1078/1438-4639-00097 11833294

[B10] Guzzi G Grandi M Cattaneo C Should amalgam fillings be removed? Lancet 2002 360 2081 10.1016/S0140-6736(02)11971-4 12504440

[B11] Guzzi G Grandi M Cattaneo C Calza S Minoia C Ronchi A Gatti A Severi G Dental amalgam and mercury levels in autopsy tissues: food for thought Am J Forensic Med Pathol 2006 27 42 45 10.1097/01.paf.0000201177.62921.c8 16501347

[B12] Levy M Schwartz S Dijak M Weber JP Tardif R Rouah F Childhood urine mercury excretion: dental amalgam and fish consumption as exposure factors Environ Res 2004 94 283 290 10.1016/j.envres.2003.07.004 15016596

[B13] Lorscheider FL Vimy MJ Summers AO Mercury exposure from "silver" tooth fillings: emerging evidence questions a traditional dental paradigm FASEB Journal 1995 9 504 508 7737458

[B14] Kingman A Albertini T Brown LJ Mercury concentrations in urine and whole blood associated with amalgam exposure in a US military population J Dent Res 1998 77 461 471 10.1177/00220345980770030501 9496919

[B15] Mortada WI Sobh MA El-Defrawy MM Farahat SE Mercury in dental restoration: is there a risk of nephrotoxicity? J Nephrol 2002 15 171 176 12018634

[B16] Nylander M Mercury in pituitary glands of dentists Lancet 1986 22 442 10.1016/S0140-6736(86)92395-0 2868360

[B17] Nylander M Weiner J Mercury and selenium concentrations and their interrelations in organs from dental staff and the general population Br J Ind Med 1991 48 729 734 1835404 10.1136/oem.48.11.729 PMC1035447

[B18] Nylander M Friberg L Lind B Mercury concentrations in the human brain and kidneys in relation to exposure from dental amalgam fillings Swed Dent J 1987 11 179 187 3481133

[B19] Pizzichini M Fonzi M Giannerini M Mencarelli M Gasparoni A Rocchi G Kaitsas V Fonzi L Influence of amalgam fillings on Hg levels and total antioxidant activity in plasma of healthy donors Sci Total Environ 2003 301 43 50 10.1016/S0048-9697(02)00291-7 12493183

[B20] Weiner JA Nylander M The relationship between mercury concentration in human organs and different predictor variables Sci Tot Environ 1993 138 101 115 10.1016/0048-9697(93)90408-X 8259485

[B21] Zimmer H Ludwig H Bader M Determination of mercury in blood, urine and saliva for the biological monitoring of an exposure from amalgam fillings in a group with self-reported adverse health effects Int J Hyg Environ Health 2002 205 205 211 10.1078/1438-4639-00146 12040918

[B22] Danscher G Hørsted-Bindsley P Rungby J Traces of mercury in organs from primates with amalgam fillings Exp Mol Pathol 1990 52 291 299 10.1016/0014-4800(90)90070-T 2115006

[B23] Galic N Prpic-Mehicic G Prester LJ Blanusa M Krnic Z Ferencic Z Dental amalgam mercury exposure in rats Biometals 1999 12 227 237 10.1023/A:1009267513632 10581685

[B24] Galic N Prpic-Mehicic G Prester LB Krnic Z Blanusa M Erceg D Elimination of mercury from amalgam in rats J Trace Elem Med Biol 2001 15 1 4 10.1016/S0946-672X(01)80018-3 11603820

[B25] Hahn LJ Kloiber R Vimy MJ Takahashi Y Lorscheider FL Dental "silver" tooth fillings: a source of mercury exposure revealed by whole-body image scan and tissue analysis FASEB Journal 1989 3 2641 2646 2636872 10.1096/fasebj.3.14.2636872

[B26] Hahn LJ Kloiber R Leininger RW Vimy M Lorscheider FL Whole-body imaging of the distribution of mercury released from dental fillings into monkey tissues FASEB Journal 1990 4 3256 3260 2227216 10.1096/fasebj.4.14.2227216

[B27] Lorscheider FL Vimy MJ Mercury exposure from "silver" fillings Lancet 1991 337 1103 10.1016/0140-6736(91)91757-L 1673530

[B28] Vimy MJ Takahashi Y Lorscheider FL Maternal-fetal distribution of mercury (203 Hg) released from dental amalgam fillings Am J Physiol 1990 258 939 945 10.1152/ajpregu.1990.258.4.R939 2331037

[B29] Heintze U Edwardsson S Derand T Birkhed D Methylation of mercury from dental amalgam and mercuric chloride by oral streptococci in vitro Scand J Dent Re 1983 91 150 152 10.1111/j.1600-0722.1983.tb00792.x 6222462

[B30] Leistevuo J Leistevuo T Helenius H Pyy L Osterblad M Huovinen P Tenovuo J Dental amalgam fillings and the amount of organic mercury in human saliva Caries Res 2001 35 163 166 10.1159/000047450 11385194

[B31] Yannai S Berdicevsky I Duek L Transformations of inorganic mercury by Candida albicans and Saccharomyces cerevisiae Appl Environ Microbiol 1991 57 245 247 2036011 10.1128/aem.57.1.245-247.1991 PMC182692

[B32] Leong CCW Syed NI Lorscheider FL Retrograde degeneration of neurite membrane structural integrity of nerve growth cones following in vitro exposure to mercury Neuro Report 2001 12 733 737 10.1097/00001756-200103260-00024 11277574

[B33] Olivieri G Brack C Muller-Spahn F Stähelin HB Herrmann M Renard P Brockhaus M Hock C Mercury induces cell cytotoxicity and oxidative stress and increases beta-amyloid secretion and tau phosphorylation in SHSY5Y neuroblastoma cells J Neurochem 2000 71 231 236 10.1046/j.1471-4159.2000.0740231.x 10617124

[B34] Olivieri G Novakovic M Savaskan E Meier F Baysang G Brockhaus M Müller-Spahn F The effects of ß-Estradiol on SHSY5Y neuroblastoma cells during heavy metal induced oxidative stress, neurotoxicity and ß-Amyloid secretion Neuroscience 2002 113 849 855 10.1016/S0306-4522(02)00211-7 12182891

[B35] Pendergrass JC Haley BE Friberg LT, Schrauzer GN Mercury-EDTA Complex Specifically Blocks Brain-Tubulin-GTP Interactions: Similarity to Observations in Alzheimer's Disease Status Quo and Perspective of Amalgam and Other Dental Materials. International Symposium Proceedings 1995 Stuttgart: Thieme Verlag 98 105

[B36] Pendergrass JC Haley BE Sigel A, Sigel H Inhibition of brain tubulin-guanosine 5'-triphosphate interactions by mercury: similarity to observations in Alzheimer's diseased brain MetalIons on Biological systems 1997 New York: Dekker 461 478 9046580

[B37] Björkman L Lundekvam BF Laegreid T Mercury in human brain, blood, muscle and toenails in relation to exposure: an autopsy study Environ Health 2007 11 6:30 10.1186/1476-069X-6-30 PMC2098763 17931423

[B38] Wenstrup D Ehmann WD Markesbery WR Trace element imbalances in isolated subcellular fractions of Alzheimer's disease brains Brain Research 1990 533 125 31 10.1016/0006-8993(90)91804-P 2085723

[B39] Ehmann WD Markesbery WR Alauddin M Hossain TIM Brubakern EH Brain trace elements in Alzheimer's disease Neurotoxicology 1986 7 197 206 3714121

[B40] Thompson CM Markesbery WR Ehmann WD Mao YX Vance DE Regional brain trace-element studies in Alzheimer's disease Neurotoxicology 1988 9 1 8 3393299

[B41] Saxe SR Wekstein MW Kryscio RJ Henry RG Cornett CR Snowdon DA Grant FT Schmitt FA Donegan SJ Wekstein DR Ehmann WD Markesbery WR Alzheimer's disease, dental amalgam and mercury J Am Dent Ass 1999 130 191 199 10036842 10.14219/jada.archive.1999.0168

[B42] Cornett CR Ehmann WD Wekstein DR Markesbery WR Trace elements in Alzheimer's disease pituitary glands Biol Trace Element Res 1998 62 107 114 10.1007/BF02820026 9630429

[B43] Braak H Neuroanatomy of Alzheimer's disease Alzheimer's Disease Review 1997 3 235 47

[B44] Mutter J Naumann J Sadaghiani C Schneider R Walach H Alzheimer Disease: Mercury as a pathogenic factor and apolipoprotein E as a moderator Neuro Endocrinol Lett 2004 25 275 283 15580166

[B45] Ask K Akesson A Berglund M Vahter M Inorganic mercury and methylmercury in placentas of Swedish women Environ Health Perspect 2002 110 523 526 10.1289/ehp.02110523 12003757 PMC1240842

[B46] Holmes AS Blaxill MF Haley BE Reduced levels of mercury in first baby haircuts of autistic children Int J Toxicol 2003 22 277 85 10.1080/10915810305120 12933322

[B47] Morgan DL Chanda SM Price HC Fernando R Liu J Brambila E O'Connor RW Beliles RP Barone S Jr Disposition of inhaled mercury vapor in pregnant rats: maternal toxicity and effects on developmental outcome Toxicol Sci 2002 66 261 273 10.1093/toxsci/66.2.261 11896293

[B48] Takahashi Y Tsuruta S Hasegawa J Kameyama Y Yoshida M Release of mercury from dental amalgam fillings in pregnant rats and distribution of mercury in maternal and fetal tissues Toxicology 2001 163 115 126 10.1016/S0300-483X(01)00390-0 11516521

[B49] Takahashi Y Tsuruta S Arimoto M Tanaka H Yoshida M Placental transfer of mercury in pregnant rats which received dental amalgam restorations Toxicology 2003 185 23 33 10.1016/S0300-483X(02)00588-7 12505442

[B50] Vahter M Akesson A Lind B Bjors U Schutz A Berglund F Longitudinal study of methylmercury and inorganic mercury in blood and urin of pregnant and lactating women, as well as in umbilical cord blood Environ Res 2000 84 186 194 10.1006/enrs.2000.4098 11068932

[B51] Yoshida M Satoh M Shimada A Yamamoto E Yasutake A Tohyama C Maternal-to-fetus transfer of mercury in metallothionein-null pregnant mice after exposure to mercury vapor Toxicology 2002 175 215 222 10.1016/S0300-483X(02)00084-7 12049849

[B52] Yoshida M Watanabe C Satoh M Yasutake A Sawada M Ohtsuka Y Akama Y Tohyama C Susceptibility of Metallothionein-Null Mice to the Behavioural Alterations Caused by Exposure to Mercury Vapour at Human-Relevant Concentration Toxicol Sci 2004 80 69 73 10.1093/toxsci/kfh138 15071173

[B53] Luglie PF Campus G Chessa G Spano G Capobianco G Fadda GM Dessole S Effect of amalgam fillings on the mercury concentration in human amniotic fluid Arch Gynecol Obstet 2005 271 138 142 10.1007/s00404-003-0578-6 14689312

[B54] Drasch G Aigner S Roider G Staiger F Lipowskyn G Mercury in human colostrum and early breast milk. Its dependence on dental amalgam and other factors J Trace Elem Med Biol 1998 12 23 27 9638609 10.1016/s0946-672x(98)80017-5

[B55] Oskarsson A Schultz A Skerfving S Hallen IP Ohlin B Lagerkvist BJ Total and inorganic mercury in breast milk in relation to fish consumption and amalgam in lactating women Arch Environ Health 1996 51 234 241 10.1080/00039896.1996.9936021 8687245

[B56] Vimy MJ Hooper DE King WW Lorscheider FL Mercury from maternal "silver" tooth fillings in sheep and human breast milk. A source of neonatal exposure Biol Trace Element Res 1997 56 143 152 10.1007/BF02785388 9164660

[B57] Waly M Olteanu H Banerjee R Choi SW Mason JB Parker BS Sukumar S Shim S Sharma A Benzecry JM Power-Charnitsky VA Deth RC Activation of methionine synthase by insulin-like growth factor and dopamine: a target for neurodevelopmental toxins and thimerosal Mol Psychiatry 2004 9 358 370 10.1038/sj.mp.4001476 14745455

[B58] Deth RC Truth revealed: New scientific discoveries regarding mercury in medicine and autism Congression Testimony before the US House of Representatives. Subcommittee in human rights and wellness 2004

[B59] Palkovicova L Ursinyova M Masanova V Yu Z Hertz-Picciotto I Maternal amalgam dental fillings as the source of mercury exposure in developing fetus and newborn J Expo Sci Environ Epidemiol 2008 18 Suppl 3 326 331 10.1038/sj.jes.7500606 17851449

[B60] Unuvar E Ahmadov H Kiziler AR Mercury levels in cord blood and meconium of healthy newborns and venous blood of their mothers: Clinical, prospective cohort study Sci Total Environ 2007 374 Suppl 1 60 70 17258795 10.1016/j.scitotenv.2006.11.043

[B61] Jedrychowski W Jankowski J Flak E Skarupa A Mroz E Sochacka-Tatara E Lisowska-Miszczyk I Szpanowska-Wohn A Rauh V Skolicki Z Kaim I Perera F Effects of prenatal exposure to mercury on cognitive and psychomotor function in one-year-old infants: epidemiologic cohort study in Poland Ann Epidemiol 2006 16 439 447 10.1016/j.annepidem.2005.06.059 16275013

[B62] Stoz F Aicham P Jovanovic S Steuer W Mayer R Ist ein generelles Amalgam-Verbot gerechtfertigt? [Is a generalized amalgam banning appropriate?] Z Geburtsh Neonat 1995 199 35 41 7725768

[B63] Hargreaves RJ Evans JG Janota I Magos L Cavanagh JB Persistant mercury in nerve cells 16 years after metallic mercury poisoning Neuropath Appl Neurobiol 1988 14 443 452 10.1111/j.1365-2990.1988.tb01336.x 3226504

[B64] Opitz H Schweinsberg F Grossmann T Wendt-Gallitelli MF Meyermann R Demonstration of mercury in the human brain and other organs 17 years after metallic mercury exposure Clin Neuropath 1996 15 139 144 8793247

[B65] Drasch G Böse-O'Reilly S Beinhoff C Roider G Maydl S The Mt. Diwata study on the Philippines 1999 - assessing mercury intoxication of the population by small scale gold mining Sci Total Environ 2001 267 151 168 10.1016/S0048-9697(00)00806-8 11286210

[B66] Drasch G Böse-O`Reilly S Maydl S Roider G Scientific comment on the German human biological monitoring values (HBM values) for mercury Int J Hyg Environ Health 2002 205 509 512 10.1078/1438-4639-00178 12455274

[B67] Drasch G Böse-O'Reilly S Maydl S Roider G Response to the letter of the Human Biomonitoring Commission Int J Hyg Environ Health 2004 207 183 184 10.1078/1438-4639-00280

[B68] Stenman S Grans L Symptoms and differential diagnosis of patients fearing mercury toxicity from amalgam fillings Scand J Work Environ Health 1997 23 59 63 9456068

[B69] Grandjean P Weihe P White R Milestone development in infants exposed to methylmercury from human milk Neurotoxicology 1995 16 27 33 7603642

[B70] Köhler W Linde K Halbach S Zilker T Kremers L Saller R Melchart D Prognos in the diagnosos of amalgam hypersensitivity: a diagnostic case-control study Forsch Komplement Med 2007 14 18 24 10.1159/000097998 17341883

[B71] WHO Mercury in Health Care Policy Paper 2005 http://www.who.int/water_sanitation_health/medicalwaste/mercurypolpaper.pdf

[B72] Viola P Cassano GB The effect of chlorine on mercury vapor intoxication. Autoradiographic study Med Lavoro 1968 59 437 44 5738990

[B73] Kishi R Doi R Fukuchi Y Satoh H Satoh T Ono A Moriwaka F Tashiro K Takahata N Subjective symptoms and neurobehavioral performances of ex-mercury miners at an average of 18 years after the cessation of chronic exposure to mercury vapor. Mercury Workers Study Group Environl Res 1993 62 289 302 10.1006/enrs.1993.1114 8344236

[B74] Mathiesen T Ellingsen DG Kjuus H Neuropsychological effects associated with exposure to mercury vapor among former chloralkali workers Scand J Work Environ Health 1999 25 342 350 10505660 10.5271/sjweh.444

[B75] Meyer-Baron M Schaeper M Seeber A A meta-analysis for neurobehavioral results due to occupational mercury exposure Arch Toxicol 2002 76 127 136 10.1007/s00204-002-0327-9 11967617

[B76] Piikivi L Hanninen H Martelin T Mantere P Psychological performance and long-term exposure to mercury vapors Scand J Work Environ Health 1984 10 35 41 6740275 10.5271/sjweh.2365

[B77] Roels H Gennart JP Lauwerys R Buchet JP Malchaire J Bernard A Surveillance of workers exposed to mercury vapour: validation of a previously proposed biological threshold limit value for mercury concentration in urine Am J Ind Med 1985 7 45 71 10.1002/ajim.4700070106 3871586

[B78] Smith PJ Langolf GD Goldberg J Effects of occupational exposure to elemental mercury on short term memory Br J Ind Med 1983 40 413 419 6626469 10.1136/oem.40.4.413 PMC1009214

[B79] Soleo L Urbano ML Petrera V Ambrosi L Effects of low exposure to inorganic mercury on psychological performance Brit J Ind Med 1990 47 105 109 10.1136/oem.47.2.105 PMC1035109 2310714

[B80] Williamson AM Teo RK Sanderson J Occupational mercury exposure and its consequences for behaviour. Int Arch Occup Environ Health 1982 50 273 286 7129652 10.1007/BF00378089

[B81] Zavariz C Glina DM Clinico-neuro-psychological evaluation of workers exposed to metallic mercury in the electric lamp industry Rev Saud Publica 1992 26 356 65 (In Portugese with English abstract) 10.1590/s0034-89101992000500010 1342526

[B82] He F Zhow X Lin B Xiung YP Chen SY Zhang SL Ru JY Deng MH Prognosis of Mercury poisoning in mercury refinery workers Ann Acad Med Singapore 1984 13 389 393 6497343

[B83] Kishi R Doi R Fukushi Y Satoh H Ono A Residual neurobehavioural effects associated with chronic exposure to mercury vapour Occup Environ Med 1994 51 35 41 10.1136/oem.51.1.35 8124461 PMC1127898

[B84] Kobal A Horvat M Prezelj M Briski AS Krsnik M Dizdarevic T Mazej D Falnoga I Stibilj V Arneric N Kobal D Osredkar J The impact of long-term past exposure to elemental mercury on antioxidative capacity and lipid peroxidation in mercury miners J Trace Elem Med Biol 2004 17 261 274 10.1016/S0946-672X(04)80028-2 15139389

[B85] Letz R Gerr F Cragle D Green R Watkins J Fidler A Residual neurologic deficits 30 years after occupational exposure to elemental mercury Neurotoxicology 2000 21 459 474 11022856

[B86] Sugita M The biological half-time of heavy metals. The existence of a third, `slowest' component Int Arch Occup Environ Health 1978 41 25 40 10.1007/BF00377797 627414

[B87] Takahata N Hayashi H Watanabe S Anso T Accumulation of mercury in the brains of two autopsy cases with chronic inorganic mercury poisoning Folia Psychiatr Neurol Jpn 1970 24 59 69 5535917 10.1111/j.1440-1819.1970.tb01457.x

[B88] Stoiber T Bonacker D Bohm K Disturbed microtubule function and induction of micronuclei by chelate complexes of mercury(II) Mutat Res 2004 563 97 106 15364276 10.1016/j.mrgentox.2004.06.009

[B89] Stoiber T Degen GH Bolt HM Unger E Interaction of mercury(II) with the microtubule cytoskeleton in IMR-32 neuroblastoma cells Toxicol Lett 2004 151 Suppl 1 99 104 10.1016/j.toxlet.2003.11.017 15177645

[B90] Thier R Bonacker D Stoiber T Interaction of metal salts with cytoskeletal motor protein systems Toxicol Lett 2003 140 75 81 10.1016/S0378-4274(02)00502-7 12676453

[B91] Duhr EF Pendergrass JC Slevin JT Haley BE HgEDTA complex inhibits GTP interactions with the E-site of brain beta-tubulin Toxicol Appl Pharmacol 1993 122 273 280 10.1006/taap.1993.1196 8212009

[B92] Pendergrass JC Haley BE Vimy MJ Winfield SA Lorscheider FL Mercury vapor inhalation inhibits binding of GTP to tubulin in rat brain: similarity to a molecular lesion in Alzheimer diseased brain Neurotoxicology 1996 18 315 324 9291481

[B93] Soares FA Farina M Santos FW Souza D Rocha JB Nogueira CW Interaction between metals and chelating agents affects glutamate binding on brain synaptic membranes Neurochem Res 2003 28 1859 1865 10.1023/A:1026175825871 14649728

[B94] Aposhian HV Morgan DL Queen HL Maiorino RM Aposhian MM Vitamin C, glutathione, or lipoic acid did not decrease brain or kidney mercury in rats exposed to mercury vapor J Toxicol Clin Toxicol 2003 41 339 347 10.1081/CLT-120022000 12870874

[B95] Nogueira CW Soares FA Nascimento PC Muller D Rocha JB 2,3-Dimercaptopropane-1-sulfonic acid and meso-2,3-dimercaptosuccinic acid increase mercury- and cadmium-induced inhibition of delta-aminolevulinate dehydratase Toxicology 2003 184 85 95 10.1016/S0300-483X(02)00575-9 12499112

[B96] Ewan KB Pamphlett R Increased inorganic mercury in spinal motor neurons follwoing chelating agents Neurotoxicology 1996 17 343 349 8856730

[B97] Harris HH Pickering IJ George GN The chemical form of mercury in fish Science 2003 301 1203 10.1126/science.1085941 12947190

[B98] Fredriksson A Dencker L Archer T Danielsson BR Prenatal coexposure to metallic mercury vapour and methylmercury produce interactive behavioural changes in adult rats Neurotoxicol Teratol 1996 18 129 134 10.1016/0892-0362(95)02059-4 8709923

[B99] Lettmeier B Böse o Reilly S Drasch G Proposal for a revised reference concentration (RFC) for mercury vapour in adults Sci Total Environ 2010 10.1016/j.scitotenv.2010.04.027 20576543

[B100] Richardson GM Environment Division of SNC-Lavalin Inc (SLE), Ottawa (Canada). Mercury exposure and risks from dental amalgam, part 1: updating exposure, reexamining reference exposure levels, and critically evaluating recent studies 2010 http://iaomt.org/articles/files/files329/Amalgam%20Risk%20Assessment%20Part%201.SLE%20reference%2010738.Final2.pdf20926115

[B101] Schubert J Riley EJ Tyler SA Combined effects in toxicology - a rapid systematic testing procedure: cadmium, mercury, and lead J Toxicol Environ Health 1978 4 763 776 10.1080/15287397809529698 731728

[B102] Haley B The relationship of toxic effects of mercury to exacerbation of the medical condition classified as alzheimer's disease http://www.fda.gov/ohrms/dockets/dailys/02/Sep02/091602/80027dd5.pdf

[B103] Ericson JE Shirahata H Patterson CC Skeletal concentrations of lead in ancient Peruvians N Engl J Med 1979 300 946 951 10.1056/NEJM197904263001703 372802

[B104] Ericson JE Smith DR Flegal AR Skeletal concentrations of lead, cadmium, zinc, and silver in ancient North American Pecos Indians Environ Health Perspect 1991 93 217 223 10.2307/3431192 1773793 PMC1568040

[B105] Drasch G Lead burden in prehistorical, historical and modern human bones Sci Total Environ 1982 24 199 231 10.1016/0048-9697(82)90001-8 7123205

[B106] Patterson CC Shirahata H Ericson JE Lead in ancient human bones and the relevance to historical developments of social problems with lead Sci Total Environ 1987 61 167 200 10.1016/0048-9697(87)90366-4 3554506

[B107] Patterson CC Shirahata H Ericson JE Natural skeletal levels of lead in Homo sapiens sapiens uncontaminated by technological lead Sci Total Environ 1991 107 205 236 10.1016/0048-9697(91)90260-L 1785050

[B108] Haley B Mercury toxicity: Genetic susceptibilities and synergistic effects Medical Veritas 2005 2 535 542 10.1588/medver.2005.02.00070

[B109] Haley B Small T Biomarkers supporting mercury toxicity as the major exacerbator of neurological illness, recent evidence via the urine prophyrin tests Medical Veritas 2006 3 1 14

[B110] Kehe K Reichl FX Durner J Walther U Hickel R Forth W Cytotoxicity of dental composite components and mercury compounds in pulmonary cells Biomaterials 2001 22 317 322 10.1016/S0142-9612(00)00184-8 11205434

[B111] Reichl FX Walther UI Durner J Kehe K Hickel R Kunzelmann KH Spahl W Hume WR Benschop H Forth W Cytotoxicity of dental composite components and mercury compounds in lung cells Dent Mater 2001 17 95 101 10.1016/S0109-5641(00)00029-4 11163377

[B112] Reichl FX Simon S Esters M Seiss M Kehe K Kleinsasser N Hickel R Cytotoxicity of dental composite (co)monomers and the amalgam component Hg(2+) in human gingival fibroblasts Arch Toxicol 2006 80 465 472 10.1007/s00204-006-0073-5 16474958

[B113] Reichl FX Esters M Simon S Seiss M Kehe K Kleinsasser N Folwaczny M Glas J Hickel R Cell death effects of resin-based dental material compounds and mercurials in human gingival fibroblast Arch Toxicol 2006 80 370 377 10.1007/s00204-005-0044-2 16691427

[B114] Walther UI Walther SC Liebl B Kehe K Hickel R Kunzelmann KH Spahl W Hume WR Benschop H Forth W Cytotoxicity of ingredients of various dental materials and related compounds in L2- and A549 cells J Biomed Mater Res 2002 63 643 649 10.1002/jbm.10384 12209911

[B115] Di Pietro A Visalli G La Maestra S Biomonitoring of DNA damage in peripheral blood lymphocytes of subjects with dental restorative fillings Mutat Res 2008 650 115 122 18178127 10.1016/j.mrgentox.2007.10.023

[B116] Schmid K Sassen A Staudenmaier R Mercury dichloride induces DNA-damage in human salivary gland tissue calls and lymphocytes Arch Toxicol 2007 1 759 767 10.1007/s00204-007-0208-3 17479252

[B117] Akiyama M Oshima H Nakamura M Genotoxicity of mercury used in chromosome aberration tests Toxicol in Vitro 2001 15 463 467 10.1016/S0887-2333(01)00051-0 11566579

[B118] Pizzichini M Fonzi M Sugherini L Fonzi L Gasparoni A Comporti M Pompella A Release of mercury from dental amalgam and its influence on salivary antioxidant activity Bull Group Int Rech Sci Stomatol Odontol 2000 42 94 100 11799733

[B119] Pizzichini M Fonzi M Sugherini L Fonzi L Comporti M Gasparoni A Pompella A Release of mercury from dental amalgam and its influence on salivary antioxidant activity Sci Total Environ 2002 284 19 25 10.1016/S0048-9697(01)00853-1 11846163

[B120] Pizzichini M Fonzi M Gasparoni A Fonzi L Comporti M Gasparoni A Pompella A Influence of amalgam fillings on Hg levels and total antioxidant activity in plasma of healthy donors Bull Group Int Rech Sci Stomatol Odontol 2001 43 62 67 11799720

[B121] Pizzichini M Fonzi M Giannerini F Mencarelli M Gasparoni A Rocchi G Kaitsas V Fonzi L Influence of amalgam fillings on Hg levels and total antioxidant activity in plasma of healthy donors Sci Total Environ 2003 301 43 50 10.1016/S0048-9697(02)00291-7 12493183

[B122] Ionescu JG Novotny J Stejskal V Lätsch A Blaurock-Busch E Eisenmann-Klein M Increased levels of transition metals in breast cancer tissue Neuro Endocrinol Lett 2006 27 36 39 16804515

[B123] Drasch G Mailänder S Schlosser C Roider G Content of non-mercury-associated selenium in human tissues Biol Trace Element Res 2000 77 219 230 10.1385/BTER:77:3:219 11204464

[B124] Mutter J Curth A Naumann J Deth R Walach H Does Inorganic Mercury Play a Role in Alzheimer's Disease? A Systematic Review and an Integrated Molecular Mechanism J Alzheimers Dis 2010 22 357 374 20847438 10.3233/JAD-2010-100705

[B125] Liebert CA Wireman J Smith T Summers AO The impact of mercury released from dental "silver" fillings on antibiotic resistances in the primate oral and intestinal bacterial flora Met Ions Biol Syst 1997 34 441 460 9046579

[B126] Lorscheider FL Vimy MJ Summers AO Zwiers H The dental amalgam mercury controversy--inorganic mercury and the CNS; genetic linkage of mercury and antibiotic resistances in intestinal bacteria Toxicology 1995 97 19 22 10.1016/0300-483X(94)02964-V 7716785

[B127] Summers AO Wireman J Vimy MJ Lorscheider FL Marshall B Levy SB Mercury released from dental "silver" fillings provokes an increase in mercury- and antibiotic-resistant bacteria in oral and intestinal floras of primates Antimicrob Agents Chemother 1993 37 825 834 8280208 10.1128/aac.37.4.825 PMC187773

[B128] Davis IJ Roberts AP Ready D Richards H Wilson M Mullany P Linkage of a novel mercury resistance operon with streptomycin resistance on a conjugative plasmid in Enterococcus faecium Plasmid 2005 54 26 38 10.1016/j.plasmid.2004.10.004 15907536

[B129] Skurnik D Ruimy R Ready D Ruppe E Bernède-Bauduin C Djossou F Guillemot D Pier GB Andremont A Is exposure to mercury a driving force for the carriage of antibiotic resistance genes? J Med Microbiol 2010 59 804 807 10.1099/jmm.0.017665-0 20339018

[B130] Leistevuo J Jarvinen H Osterblad M Leistevuo T Huovinen P Tenovuo J Resistance to mercury and antimicrobial agents in Streptococcus mutans isolates from human subjects in relation to exposure to dental amalgam fillings Antimicrob Agents Chemother 2000 44 456 457 10.1128/AAC.44.2.456-457.2000 10639385 PMC89706

[B131] Pike R Lucas V Stapleton P Gilthorpe MS Roberts G Rowbury R Richards H Mullany P Wilson M Prevalence and antibiotic resistance profile of mercury-resistant oral bacteria from children with and without mercury amalgam fillings J Antimicrob Chemother 2002 49 777 783 10.1093/jac/dkf019 12003971

[B132] Wireman J Liebert CA Smith T Summers AO Association of mercury resistance with antibiotic resistance in the gram-negative fecal bacteria of primates Appl Environ Microbiol 1997 63 4494 4503 9361435 10.1128/aem.63.11.4494-4503.1997 PMC168768

[B133] Harris HH Vogt S Eastgate H Legnini DG Hornberger B Cai Z Lai B Lay PA Migration of mercury from dental amalgam through human teeth J Synchrotron Radiat 2008 15 123 128 10.1107/S0909049507061468 18296776

[B134] Weidinger S Kramer U Dunemann L Mohrenschlager M Ring J Behrendt H Body burden of mercury is associated with acute atopic eczema and total IgE in children from southern Germany J Allergy Clin Immunol 2004 114 457 459 10.1016/j.jaci.2004.04.011 15341030

[B135] Berlin M Mercury in dental-filling materials - an updated risk analysis in environmental medical terms The Dental Material Comission - Care and Consideration 2003 Sweden

[B136] Dunsche A Frank M Luttges J Açil Y Brasch J Christophers E Springer IN Lichenoid reactions of murine mucosa associated with amalgam Br J Dermatol 2003 148 741 748 10.1046/j.1365-2133.2003.05229.x 12752133

[B137] Dunsche A Kastel I Terheyden H Springer I Christophers E Brasch J Oral lichenoid reactions associated with amalgam: improvement after amalgam removal Br J Dermatol 2003 148 70 76 10.1046/j.1365-2133.2003.04936.x 12534597

[B138] Martin M Broughton S Drangsholt M Oral lichen planus and dental materials: a case-control study Contact Dermatitis 2003 48 331 336 10.1034/j.1600-0536.2003.00146.x 14531872

[B139] Wong L Freeman S Oral lichenid lesions (OLL) and mercury in amalgam fillings Contact Dermatitis 2003 48 74 79 10.1034/j.1600-0536.2003.480204.x 12694209

[B140] Guttman-Yassky E Weltfriend S Bergman R Resolution of orofacial granulomatosis with amalgam removal J Eur Acad Dermatol Venerol 2003 17 344 347 10.1046/j.1468-3083.2003.00793.x 12702083

[B141] Guarneri F Marini H Perioral dermatitis after dental filling in a 12-year-old girl: involvement of cholinergic system in skin neuroinflammation? ScientificWorldJournal 2008 8 157 163 10.1100/tsw.2008.31 18264633 PMC5848648

[B142] Pigatto PD Brambilla L Guzzi G Mercury pink exanthem after dental amalgam placement J Eur Acad Dermatol Venereol 2008 22 377 378 10.1111/j.1468-3083.2007.02332.x 18269611

[B143] Bartova J Prochazkova J Kratka Z Benetkova K Venclikova Z Sterzl I Dental amalgam as one of the risk factors in autoimmune diseases Neuro Endocrinol Lett 2003 24 65 67 12743535

[B144] Hultman P Johansson U Turley S Lindh U Enestrom S Pollard K Adverse immunological effects and autoimmunity induced by dental amalgam and alloy in mice FASEB Journal 1994 8 1183 1190 7958626 10.1096/fasebj.8.14.7958626

[B145] Hultman P Lindh U Horsted-Binslev P Activation of the immune system and systemic immune-complex deposits in Brown Norway rats with dental amalgam restorations J Dent Res 1998 77 1415 1425 10.1177/00220345980770060601 9649170

[B146] Pollard KM Pearson DL Hultman P Deane TN Lindh U Kono DH Xenobiotic acceleration of idiopathic systemic autoimmunity in lupus-prone bxbs mice Environ Health Persp 2001 109 27 33 10.2307/3434917 PMC1242047 11171521

[B147] Prochazkova J Sterzl I Kucerova H Bartova J Stejskal VDM The beneficial effect of amalgam replacement on health in patients with autoimmunity Neuro Endocrinol Lett 2004 25 211 218 15349088

[B148] Stejskal J Stejskal VD The role of metals in autoimmunity and the link to neuroendocrinology Neuro Endocrinol Lett 1999 20 351 364 11458198

[B149] Stejskal VD Danersund A Lindvall A Metal-specific lymphocytes: biomarkers of sensitivity in man Neuro Endocrinol Lett 1999 20 289 298 11460087

[B150] Sterzl I Procházková J Hrdá P Bártová J Matucha P Stejskal VDM Mercury and nickel allergy: risk factors in fatigue and autoimmunity Neuro Endocrinol Lett 1999 20 221 228 11462117

[B151] Via CS Nguyen P Niculescu F Papadimitriou J Hoover D Silbergeld EK Low-dose exposure to inorganic mercury accelerates disease and mortality in acquired murine lupus Environ Health Perspect 2003 111 1273 1277 10.1289/ehp.6064 12896845 PMC1241605

[B152] Sterzl I Procházková J Hrda P Matucha P Bartova J Stejskal V Removal of dental amalgam decreases anto-TPO and anti-Tg autoantibodies in patients with autoimmune thyroiditis Neuro Endocrinol Lett 2006 5 27(Suppl 1 25 30 16804512

[B153] Kazantzis G Mercury exposure and early effects: an overview Med Lav 2002 93 139 147 12197264

[B154] Rampersad GC Suck G Sakac D Fahim S Foo A Denomme GA Langler RF Branch DR Chemical compounds that target thiol-disulfide groups on mononuclear phagocytes inhibit immune mediated phagocytosis of red blood cells Transfusion 2005 45 384 393 10.1111/j.1537-2995.2005.04241.x 15752156

[B155] Bartram F Donate HP Müller KE Bückendorf CH Ohnsorge P Huber W von Baehr V Significance of the patch test and the lymphocyte transformation test in the diagnostic of type IV-sensitazion J Lab Med 2006 30 101 106

[B156] Venclíková Z Benada O Bártová J Joska L Mrklas L Procházková J Stejskal V Podzimek S In vivo effects of dental casting alloys Neuro Endocrinol Lett 2006 27 Suppl 1 61 68 16892010

[B157] Valentine-Thon E Schiwara HW Validity of MELISA for metal sensitivity testing Neuro Endocrinol Lett 2003 24 50 55 12743534

[B158] Valentine-Thon E Sandkamo M Müller K Guzzi G Hartmann T Metallsensibilisierung: Nachweis, Validierung und Verlaufskontrolle mittels Lymphozyten-Transformations-Test (LTT-Melisa^®^) Zs f Orthomol Med 2005 1 12 15

[B159] Valentine-Thon E Muller KE Guzzi G Kreisel S Ohnsorge P Sandkamp M LTT-MELISA^® ^is clinically relevant for detecting and monitoring metal sensitivity Neuro Endocrinol Lett 2006 27 Suppl1 17 24 17261998

[B160] Yaqob A Danersund A Stejskal VD Lindvall A Hudecek R Lindh U Metal-specific lymphocyte reactivity is downregulated after dental metal replacement Neuro Endocrinol Lett 2006 27 189 197 16648791

[B161] Lindh U Hudecek R Dandersund A Eriksson S Lindvall A Removal of dental amalgam and other metal alloys supported by antioxidant therapy alleviates symptoms and improves quality of life in patients with amalgam-associated ill health Neuro Endocrinol Lett 2002 23 459 482 12500173

[B162] Stejskal VD Diagnosis and treatment of metal-induced side effects Neuro Endocrinol Lett 2006 27 Suppl 1 7 16 17261999

[B163] Wortberg W Intrauterine Fruchtschädigung durch Schwermetallbelastung der Mutter Umwelt Medizin Gesellschaft 2006 19 274 280

[B164] Houston MC The role of mercury and cadmium heavy metals in vascular disease, hypertension, coronary heart disease, and myocardial infarction Altern Ther Health Med 2007 13 128 133, 17405690

[B165] Frustaci A Magnavita N Chimenti C Cladarulo M Sabbioni E Pietra R Marked elevation of myocardial trace elements in idiopathic dilated cardiomyopathy compared with secondary cardiac dysfunction J Am Coll Cardiol 1999 33 1578 1583 10.1016/S0735-1097(99)00062-5 10334427

[B166] Dodes JE The amalgam controversy. An evidence-based analysis J Am Dent Assoc 2001 132 348 356 11258092 10.14219/jada.archive.2001.0178

[B167] Boyd ND Benediktsson H Vimy MJ Hooper DE Lorscheider FL Mercury from dental "silver" tooth fillings impairs sheep kidney function Am J Physiol 1991 261 1010 1014 10.1152/ajpregu.1991.261.4.R1010 1928419

[B168] Trachtenberg F Barregård L The effect of age, sex, and race on urinary markers of kidney damage in children Am J Kidney Dis 2007 50 938 945 10.1053/j.ajkd.2007.08.014 18037094

[B169] Mutter J Naumann J Sadaghiani C Walach H Quecksilber und die Alzheimer-Erkrankung Fortschr Neuro Psychiat 2007 75 528 538 10.1055/s-2007-959237 17628833

[B170] Mutter J Naumann J Guethlin C Comments on the article "the toxicology of mercury and its chemical compounds" by Clarkson and Magos (2006) Crit Rev Toxicol 2007 37 537 549 10.1080/10408440701385770 17661216

[B171] Carpenter DO Effects of metals on the nervous system of humans and animals Int J Occup Med Environ Health 2001 14 209 218 11764847

[B172] Dantzig PI Parkinson's disease, macular degeneration and cutaneous signs of mercury toxicity J Occup Environ Med 2006 48 656 16832218 10.1097/01.jom.0000228351.74230.52

[B173] Finkelstein Y Vardi J Kesten MM Hod I The enigma of parkinsonism in chronic borderline mercury intoxication, resolved by challenge with penicillamine Neurotoxicology 1996 17 291 295 8784840

[B174] Gorell JM Rybicki BA Johnson C Peterson EL Occupational metal exposures and the risk of Parkinson's disease Neuroepidemiology 1999 18 303 308 10.1159/000026225 10545782

[B175] Miller K Ochudto S Opala G Smolicha W Siuda J Parkinsonism in chronic occupational metallic mercury intoxication Neurol Neurochir Pol 2003 37 31 38 15098329

[B176] Ngim CH Devathasan G Epidemiologic study on the association between body burden mercury level and idiopathic Parkinson's disease Neuroepidemiology 1989 8 128 141 10.1159/000110175 2725805

[B177] Ohlson CG Hogstedt C Parkinson's disease and occupational exposure to organic solvents, agricultural chemicals and mercury - a case-referent study Scand J Work Environ Health 1981 7 252 256 7347910 10.5271/sjweh.2549

[B178] Rybicki BA Johnson CC Uman J Gorell JM Parkinson's disease mortality and the industrial use of heavy metals in Michigan Mov Disord 1993 8 87 92 10.1002/mds.870080116 8419812

[B179] Seidler A Hellenbrand W Robra BP Possible environmental, occupational, and other etiologic factors for Parkinson's disease: a case-control study in Germany Neurology 1996 46 1275 84 8628466 10.1212/wnl.46.5.1275

[B180] Uversky VN Li J Fink AL Metal-triggered structural transformations, aggregation, and fibrillation of human alpha-synuclein. A possible molecular NK between Parkinson's disease and heavy metal exposure J Biol Chem 2001 276 44284 44296 10.1074/jbc.M105343200 11553618

[B181] Harakeh S Sabra N Kassak K Doughan B Sukhn C Mercury and arsenic levels among Lebanese dentists: a call for action Bull Environ Contam Toxicol 2003 70 629 635 10.1007/s00128-003-0031-3 12677371

[B182] Tezel H Ertas OS Ozata F Erakin C Kayali A Blood mercury levels of dental students and dentists at a dental school Br Dent J 2001 191 449 452 10.1038/sj.bdj.4801205a 11720018

[B183] Aydin N Karaoglanoglu S Yigit A Keles MS Kirpinar I Seven N Neuropsychological effects of low mercury exposure in dental staff in Erzurum, Turkey Int Dent J 2003 53 85 91 12731695 10.1111/j.1875-595x.2003.tb00664.x

[B184] Bittner ACJ Echeverria D Woods JS Behavioral effects of low-level exposure to HgO among dental professional: a cross-study evaluation of psychomotor effects Neuortoxicol Teratol 1998 17 161 168

[B185] Echeverria D Heyer NJ Martin MD Naleway C Woods JS Bittner ACJ Behavioral effects of low-level exposure to elemental Hg among dentists Neurotoxicol Teratol 1995 17 161 168 10.1016/0892-0362(94)00049-J 7760775

[B186] Echeverria D Woods JS Heyer N Rohlman DS Farin FM Bittner AC Jr Li T Garabedian C Chronic low-level mercury exposure, BDNF polymorphism and associations with cognitive and motor function Neurotoxicol Teratol 2005 27 781 796 10.1016/j.ntt.2005.08.001 16301096

[B187] Heyer NJ Echeverria D Bittner AJ Farin FM Garabedian CC Woods JS Chronic low-level mercury exposure, BDNF polymorphism, and associations with self-reported symptoms and mood Toxicol Sci 2004 81 354 363 10.1093/toxsci/kfh220 15254338

[B188] Heyer NJ Bittner AJ Echerverria D Woods J A cascade analysis of the interaction of mercury and coproporphyrinogen-oxidase (CPOX) polymorphism on the heme biosynthetic pathway and porphyrin production Toxicol Lett 2006 161 159 166 10.1016/j.toxlet.2005.09.005 16214298

[B189] Gonzalez-Ramirez D Maiorino RM Zuniga-Charles M Sodium 2,3-dimercaptopropane-1-sulfonate challenge test for mercury in humans: II. Urinary mercury, porphyrins and neurobehavioral changes of dental workers in Monterrey, Mexico J Pharmacol Exp Ther 1995 272 264 274 7815341

[B190] Langworth S Sallsten G Barregard L Cynkier I Lind ML Soderman E Exposure to mercury vapor and impact on health in the dental profession in Sweden J Dent Res 1997 76 1397 1404 10.1177/00220345970760071001 9207773

[B191] Moen BE Hollund BE Riise T Neurological symptoms among dental assistants: a cross-sectional study J Occup Med Toxicol 2008 18 3 10 10.1186/1745-6673-3-10 PMC2427043 18485237

[B192] Ngim CH Foo SC Boey KW Jeyaratnam J Chronic neurobehavioral effects of elemental mercury in dentists Br J Ind Med 1992 M49 782 790 10.1136/oem.49.11.782 PMC1039326 1463679

[B193] Ritchie KA Macdonald EB Hammersley R O'Neil JM McGowan DA Dale IM Wesnes K A pilot study of the effect of low level exposure to mercury on the health of dental surgeons J Occup Environ Med 1995 52 813 817 10.1136/oem.52.12.813 PMC1128382 8563844

[B194] Ritchie KA Gilmour WH Macdonald EB Burke FJ McGowan DA Dale IM Hammersley R Hamilton RM Binnie V Collington D Health and neuropsychological functioning of dentists exposed to mercury J Occup Environ Med 2002 59 287 293 10.1136/oem.59.5.287 PMC1740287 11983843

[B195] Uzzell BP Oler J Chronic low-level mercury exposure and neuropsychological functioning J Clin Exp Neuropsychol 1986 8 581 593 10.1080/01688638608405177 3805254

[B196] Urban P Lukas E Nerudova J Cabelkova Z Cikrt M Neurological and electrophysiological examinations on three groups of workers with different levels of exposure to mercury vapors Eur J Neurol 1999 6 571 577 10.1046/j.1468-1331.1999.650571.x 10457390

[B197] Nadorfy-Lopez E Torres SH Finol H Mendez M Bello B Skeletal muscle abnormalities associated with occupational exposure to mercury vapors Hist Histopath 2000 15 673 682 10.14670/HH-15.673 10963110

[B198] Rowland A Baird D Weinberg C Shore D Shy C Wilcox A The effect of occupational exposure to the mercury vapour on the fertility of female dental assistants Occup Environ Med 1994 51 28 34 10.1136/oem.51.1.28 8124459 PMC1127897

[B199] Lindbohm ML Ylöstalo P Sallmen M Occupational exposure in dentistry and miscarriage Occup Environ Med 2007 64 127 133 10.1136/oem.2005.026039 17053021 PMC2078431

[B200] Jones L Bunnell J Stillman J A 30-year follow-up of residual effects on New Zealand School Dental Nurses, from occupational mercury exposure Hum Exp Toxicol 2007 26 367 374 10.1177/0960327107076824 17615119

[B201] Echeverria D Woods JS Heyer NJ Rohlman D Farin FM Li T Garabedian CE The association between a genetic polymorphism of coproporphyrinogen oxidase, dental mercury exposure and neurobehavioral response in humans Neurotoxicol Teratol 2006 28 39 48 10.1016/j.ntt.2005.10.006 16343843

[B202] Gerhard I Monga B Waldbrenner A Runnebaum B Heavy metals and fertility. J Toxicol Environ Health 1998 54 593 611 9726782 10.1080/009841098158638

[B203] Gerhard I Waibel S Daniel V Runnebaum B Impact of heavy metals on hormonal and immunological factors in women with repeated miscarriages Hum Reprod Update 1998 4 301 309 10.1093/humupd/4.3.301 9741713

[B204] Gerhard I Runnebaum B The limits of hormone substitution in pollutant exposure and fertility disorders Zentralbl Gynaekol 1992 114 593 602 1285483

[B205] Sheiner EK Sheiner E Hammel RD Potashnik G Carel R Effect of occupational exposures on male fertility: literature review Ind Health 2003 41:5 5 62 10.2486/indhealth.41.55 12725464

[B206] Podzimek S Prochazkova J Pribylova L Bártová J Ulcová-Gallová Z Mrklas L Stejskal VD Effect of heavy metals on immune reactions in patients with infertility Cas Lek Cesk 2003 142 285 288 12920793

[B207] Podzimek S Prochazkova J Bultasova L Bartova J Ulcova-Gallova Z Mrklas L Stejskal VD Sensitization to inorganic mercury could be a risk factor for infertility Neuro Endocrinol Lett 2005 26 277 282 16136024

[B208] Ahlrot-Westerlund B Mercury in cerebrospinal fluid in multiple sclerosis Swed J Biol Med 1989 1 6 7

[B209] Craelius W Comperative epidemiology of multiple sclerosis and dental caries J Epidemiol Comm Health 1978 32 155 165 10.1136/jech.32.3.155 PMC1060938 711974

[B210] McGrother C Dugmore C Phillips M Raymond N Garrick P Baird W Multiple sclerosis, dental caries and fillings: a case-control study Br Dent J 1999 187 261 264 10.1038/sj.bdj.4800255a 10520544

[B211] Baasch E Theoretical considerations on the etiology of multiple sclerosis. Is multiple sclerosis a mercury allergy? Schweiz Arch Neurol Neurochir Psychiatr 1966 98 1 19 5985125

[B212] Ingalls T Epidemiology, etiology and prevention of multiple sclerosis. Hypothesis and fact Am J Forensic Med Pathol 1983 4 55 61 10.1097/00000433-198303000-00006 6837537

[B213] Ingalls T Endemic clustering of multiple sclerosis in time and place, 1934-1984. Confirmation of a hypothesis Am J Forensic Med Pathol 1986 7 3 8 10.1097/00000433-198603000-00002 3728417

[B214] Issa Y Watts D Duxbury A Brunton P Watson M Waters C Mercuric chloride: toxicity and apoptosis in a human oligodendroglial cell line Biomaterials 2003 24 981 987 10.1016/S0142-9612(02)00436-2 12504520

[B215] Siblerud RL A comparison of mental health of multiple sclerosis patients with silver/mercury dental fillings and those with fillings removed Psychol Rep 1992 70 1139 1151 10.2466/PR0.70.4.1139-1151 1496084

[B216] Siblerud RL Kienholz E Motl J Evidence that mercury from silver dental fillings may be an etiological factor in smoking Toxicol Lett 1993 68 307 310 10.1016/0378-4274(93)90022-P 8516784

[B217] Huggins HA Levy TE Cerebrospinal fluid protein changes in multiple sclerosis after dental amalgam removal Altern Med Rev 1998 4 295 300 9727079

[B218] Bates M Fawcett J Garrett N Cutress T Kjellstrom T Related articles, health effects of dental amalgam exposure: a retrospective cohort study Int J Epidemiol 2004 33 894 902 10.1093/ije/dyh164 15155698

[B219] Bates MN Mercury amalgam dental fillings: an epidemiologic assessment Int J Hyg Environ Health 2006 209 Suppl 4 309 316 10.1016/j.ijheh.2005.11.006 16448848

[B220] Aminzadeh KK Etminan M Dental amalgam and multiple sclerosis: a systematic review and meta-analysis J Public Health Dent 2007 67 64 66 10.1111/j.1752-7325.2007.00011.x 17436982

[B221] Pamphlett R Coote P Entry of low doses of mercury vapor into the nervous system Neurotoxicology 1998 19 39 47 9498219

[B222] Pamphlett R Slater M Thomas S Oxidative damage to nuclic acids in motor neurons containing mercury J Neurol Sci 1998 159 121 126 10.1016/S0022-510X(98)00161-0 9741394

[B223] Pamphlett R Waley P Motor neuron uptake of low dose inorganic mercury J Neurol Sci 1996 135 63 67 10.1016/0022-510X(95)00258-4 8926498

[B224] Praline J Guennoc AM Limousin N Hallak H deToffol B Corcia P ALS and mercury intoxication: a relationship? Clin Neurol Neurosurg 2007 109 Suppl 10 880 883 10.1016/j.clineuro.2007.07.008 17719172

[B225] Stankovic R Atrophy of large myelinated motor axons and declining muscle grip strength following mercury vapour inhalation in mice Inhal Toxicol 2006 18 57 69 10.1080/08958370500282902 16326402

[B226] Albrecht J Matya E Glutamate: a potential mediator of inorganic mercury neurotoxicity Metab Brain Dis 1996 11 175 184 10.1007/BF02069504 8776719

[B227] Adams C Ziegler D Lin J Mercury intoxication simulating amyotrophic lateral sclerosis JAMA 1983 250 642 643 10.1001/jama.250.5.642 6864963

[B228] Schwarz S Husstedt I Bertram H Kuchelmeister K Amyotrophic lateral sclerosis after accidental injection of mercury J Neurol Neurosurg Psychiatry 1996 60 698 10.1136/jnnp.60.6.698 8648348 PMC1073965

[B229] Redhe O Pleva J Recovery from amyotrophic lateral sclerosis and from allergy after removal of dental amalgam fillings Int J Risk Saf Med 1994 4 229 236 10.3233/JRS-1994-4307 23511261

[B230] Engel P Beobachtungen über die Gesundheit vor und nach Amalgamentfernung. [Observations on health before and after removing dental amalgam] Schweiz Monatsschr Zahnm 1998 108 2 14 9776669

[B231] Godfrey ME Wojcik DP Krone CA Apolipoprotein E genotyping as a potential biomarker for mercury neurotoxicity J Alz Dis 2003 5 189 195 10.3233/jad-2003-5303 12897404

[B232] Siblerud RL The relationship between mercury from dental amalgam and mental health Am J Psychother 1989 43 575 587 2618948 10.1176/appi.psychotherapy.1989.43.4.575

[B233] Siblerud RL Motl J Kienholz E Psychometric evidence that mercury from silver dental fillings may be an etiological factor in depression, excessive anger, and anxiety Psychol Rep 1994 74 67 80 8153237 10.2466/pr0.1994.74.1.67

[B234] Wojcik DP Godfrey ME Haley B Mercury toxicity presenting as chronic fatigue, memory impairment and depression: diagnosis, treatment, susceptibility, and outcomes in a New Zealand general practice setting (1994-2006) Neuro Endocrinol Lett 2006 27 415 423 16891999

[B235] Marcusson J Psychological and somatic subjective as a result of dermatological patch testing with metallic mercury and phenyl mercuric acetate Toxicol Lett 1996 84 113 122 10.1016/0378-4274(95)03623-7 8614905

[B236] Marcusson J Jarstrand C Oxidative metabolism of neutrophils in vitro and human mercury intolerance Toxicol in Vitro 1998 12 383 388 10.1016/S0887-2333(98)00018-6 20654420

[B237] Marcusson J The frequency of mercury intolerance in patients with chronic fatigue syndrome and healthy controls Contact Dermatitis 1999 41 60 61 10.1111/j.1600-0536.1999.tb06225.x 10416724

[B238] Woods J Martin Naleway CA Echeverria D Urinary porphyrin profiles as a biomarker of mercury exposure: studies on dentists with occupational exposure to mercury vapor J Toxicol Environ Health 1993 40 235 46 10.1080/15287399309531791 8230299

[B239] Nataf R Skorupka C Amet L Lam A Springbett A Lathe R Porphyrinuria in childhood autistic disorder: implications for environmental toxicity Toxicol Appl Pharmacol 2006 214 99 108 10.1016/j.taap.2006.04.008 16782144

[B240] Geier DA Geier MR A prospective assessment of porphyrins in autistic disorders: a potential marker for heavy metal exposure Neurotox Res 2006 10 57 64 10.1007/BF03033334 17000470

[B241] Geier DA Geier MR A meta-analysis epidemiological assessment of neurodevelopmental disorders following vaccines administered from 1994 through 2000 in the United States Neuro Endocrinol Lett 2006 27 401 413 16807526

[B242] Woods JS Echeverria D Heyer NJ Simmonds PL Wilkerson J Farin FM The association between genetic polymorphisms of coproporphyrinogen oxidase and an atypical porphyrinogenic response to mercury exposure in humans Toxicol Appl Pharmacol 2005 206 113 120 10.1016/j.taap.2004.12.016 15967199

[B243] Atamna H Frey WH A Role for heme in Alzheimer's disease: Heme binds amyolid β and has altered metabolism PNAS 2004 101 Suppl 30 153 158 15263070 10.1073/pnas.0404349101 PMC503755

[B244] Stewart WF Schwartz BS Simon D Kelsey K Todd AC ApoE genotype, past adult lead exposure, and neurobehavioral function Environ Health Perspect 2002 110 501 505 10.1289/ehp.02110501 12003753 PMC1240838

[B245] Mutter J Naumann J Schneider R Walach H Haley B Mercury an autism: Accelerating evidence? Neuro Endocrinol Lett 2005 26 431 437 16264412

[B246] Mutter J Naumann J Walach H Daschner F Amalgam: Eine Risikobewertung unter Berücksichtigung der neuen Literatur bis 2005 Gesundheitswesen 2005 67 204 216 10.1055/s-2005-857962 15789284

[B247] Mutter J Naumann J Sadaghiani C Walach H Drasch G Mercury an autism: Response to the letter of K.E.v. Muehlendahl Int J Hyg Environ Health 2005 208 437 438 10.1016/j.ijheh.2005.06.001

[B248] Kidd R Results of dental amalgam removal and mercury detoxification using DMPS and neural therapy Altern Ther Health 2000 6 49 55 10895513

[B249] Lindforss H Marqvardsen O Olsson S Henningson M Effekter på hälsan efter avlägsnandet av amalgamfyllingar. Enodontologisk, medicinsk och psykosomatisk studie Tandläkartidningen 1994 86 205 211

[B250] Lygre GB Gjerdet NR Bjorkman L Patients' choice of dental treatment following examination at a specialty unit for adverse reactions to dental materials Acta Odontol Scand 2004 62 258 263 10.1080/00016350410001694 15841812

[B251] Stomberg R Langworth S Berlin M. Sweden Mercury in dental-filling materials - an updated risk analysis in environmental medical terms. The dental Material Commission - Care and Consideration 2003 19 (1998):

[B252] Faustman EM Silbernagel SM Fenske RA Burbacher T Ponce RA Mechanisms underlying children's susceptibility to environmental toxicants Environ Health Perspect 2000 108 13 21 10.2307/3454629 10698720 PMC1637781

[B253] Cheuk DK Wong V Attention-deficit hyperactivity disorder and blood mercury level: a case control study in Chinese children Neuropediatrics 2006 37 234 240 10.1055/s-2006-924577 17177150

[B254] Desoto MC Hitlan RT Blood levels of mercury are related to diagnosis of autism: a reanalysis of an important data set J Child Neurol 2007 22 1308 1311 10.1177/0883073807307111 18006963

[B255] Adams JB Romdalvik J Ramanujam VM Legator MS Mercury, lead, and zinc in baby teeth of children with autism versus controls J Toxicol Environ Health 2007 70 1046 1051 10.1080/15287390601172080 17497416

[B256] Evans TA Siedlak SL Lu L The autistic phenotype exhibits remarkably localized modification of brain protein by products of free radical-induced lipid oxidation Am J Biochem Biotechnol 2008 4 61 72 10.3844/ajbbsp.2008.61.72

[B257] Lopez-Hurtado E Prieto JJ A microscopic study of language-related cortex in autism Am J Biochem Biotechnol 2008 4 130 145 10.3844/ajbbsp.2008.130.145

[B258] Sajdel-Sulkowska EM Lipinski B Windom H Audhya T McGinnis W Oxidative stress in autism: elevated cerebellar 3-nitrotyrosine levels Am J Biochem Biotechnol 2008 4 73 84 10.3844/ajbbsp.2008.73.84

[B259] Bradstreet J Geier D Kartzinel J Adams J Geier M A case-control study of mercury burden in children with autistic spectrum disorders J Am Phys Surg 2003 8 76 79

[B260] Geier DA Geier MR A case series of children with apparent mercury toxic encephalopathies manifesting with clinical symptoms of regressive autistic disorders J Toxicol Environ Health 2007 70 837 851 10.1080/15287390701212141 17454560

[B261] Geier DA Geier MR A prospective study of mercury toxicity biomarkers in autistic spectrum disorders. J Toxicol Environ Health 2007 70 1723 1730 17885929 10.1080/15287390701457712

[B262] Adams JB Baral M Geis E Mitchell J Ingram J Hensley A Zappia I Newmark S Gehn E Rubin RA Mitchell K Bradstreet J El-Dahr J Safety and efficacy of oral DMSA therapy for children with autism spectrum disorders: part B - behavioral results BMC Clin Pharmacol 2009 9 17 10.1186/1472-6904-9-17 19852790 PMC2770991

[B263] Geier DA Kern JK Geier MR A prospective study of prenatal mercury exposure from maternal dental amalgams and autism severity Acta Neurobiol Exp 2009 69 189 197 10.55782/ane-2009-1744 19593333

[B264] Geier DA Mumper E Gladfelter B Coleman L Geier MR Neurodevelopmental Disorders, Maternal Rh-Negativity, and Rho(D) Immune Globulins: A Multi-Center Assessment Neuro Endocrinol Lett 2008 29 272 280 18404135

[B265] Hornig M Chian D Lipkin W Neurotoxic effects of postnatal thimerosal are mouse strain dependent Mol Psychiatry 2004 9 833 845 10.1038/sj.mp.4001529 15184908

[B266] Amin-Zaki L Majeed MA Greenwood MR Elhassani SB Clarkson TW Doherty RA Methylmercury poisoning in the Iraqi suckling infant: a longitudinal study over five years J Appl Toxicol 1981 1 210 214 10.1002/jat.2550010405 6892222

[B267] Counter SA Buchanan LH Ortega F Laurell G Elevated blood mercury and neuro-otological observations in children of the Ecuadorian gold mines J Toxicol Environ Health 2002 65 149 163 10.1080/152873902753396785 11820503

[B268] Debes F Budtz-Jorgensen E Weihe P White RF Grandjean P Impact of prenatal methylmercury exposure on neurobehavioral function at age 14 years Neurotoxicol Teratol 2006 28 363 75 10.1016/j.ntt.2006.02.004 16647838 PMC1543702

[B269] Palmer R Blanchard S Stein Z Mandell D Miller C Environmental mercury release, special education rates and autism disorder: an ecological study of Texas Health&Place 2006 12 203 209 10.1016/j.healthplace.2004.11.005 16338635

[B270] Rury J Links between environmental mercury special education and autism in Louisiana PhD thesis 2006 Louisiana State University, Baton Rouge (LA)

[B271] Windham GC Zhang L Gunier R Croen LA Grether JK Autism spectrum disorders in relation to distribution of hazardous air pollutants in the San Francisco Bay area Environ Health Perspect 2006 114 1438 1444 10.1289/ehp.9120 16966102 PMC1570060

[B272] James S Cutler P Melnyk S Jernigan S Janak L Gaylor DW Neubrander JA Metabolic biomarkers of increased oxidative stress and impaired methylation capacity in children with autism Am J Clin Nutr 2004 80 1611 1617 15585776 10.1093/ajcn/80.6.1611

[B273] James SJ Slikker W Melnyk S New E Pogribna M Jernigan S Thimerosal neurotoxicity is associated with glutathione depletion: protection with glutathione precursors Neurotoxicology 2005 26 1 8 10.1016/j.neuro.2004.07.012 15527868

[B274] Autism Research Institute Treatment options for mercury/metal toxicity in autism and related developmental disabilities http://autism.asu.edu/TreatmentOptions.pdf

[B275] Zahir F Rizwi SJ Haq SK Khan RH Low dose mercury toxicity and human health Environ Toxicol Pharmacol 2005 20 351 360 10.1016/j.etap.2005.03.007 21783611

[B276] Bjorkman L Pedersen NL Lichtenstein P Physical and mental health related to dental amalgam fillings in Swedish twins Community Dent Oral Epidemiol 1996 24 260 267 10.1111/j.1600-0528.1996.tb00856.x 8871034

[B277] Bellinger DC Needleman HL Intellectual impairment and blood lead levels N Eng J Med 2003 349 500 502 10.1056/NEJM200307313490515 12890850

[B278] DeRouen TA Martin MD Leroux BG Neurobehavioral effects of dental amalgam in children: a randomized clinical trial JAMA 2006 295 Suppl 15 1784 1792 10.1001/jama.295.15.1784 16622140

[B279] Buyske S Williams TA Mars AE Stenroos ES Ming SX Wang R Sreenath M Factura MF Reddy C Lambert GH Johnson WG Analysis of case-parent trios at a locus with a deletion allele: association of GSTM1 with autism BMC Genet 2006 7 8 10.1186/1471-2156-7-8 16472391 PMC1382247

[B280] Chew CL Soh G Lee AS Yeoh TS Long-term dissolution of mercury from a non-mercury-releasing amalgam Clin Prev Dent 1991 13 Suppl 3 5 7 1860296

[B281] UNEP (United Nations Environment Programm Chemicals) Global Mercury Assessment 2002 http://www.chem.unep.ch/mercury/Report/GMA-report-TOC.htm

[B282] Laks DR Assessment of chronic mercury exposure within the U.S. population, National Health and Nutrition Examination Survey, 1999-2006 Biometals 2009 [Epub ahead of print] 19697139 10.1007/s10534-009-9261-0

[B283] Hylander L Goodsite M Environmental costs of the mercury pollution Sci Total Environ 2006 368 352 370 10.1016/j.scitotenv.2005.11.029 16442592

[B284] Hylander L Lindvall A Gahnberg L High mercury emissions from dental clinics despite amalgam separators Sci Total Environ 2006 362 74 84 10.1016/j.scitotenv.2005.06.008 16054673

[B285] Hylander L Lindvall A Uhrberg R Gahnberg L Lindh U Mercury recovery in situ of four different dental amalgam separators Sci Total Environ 2006 366 320 336 10.1016/j.scitotenv.2005.07.007 16182343

[B286] Bender M Taking a bite out of dental mercury pollution. New England zero Mercury Campaign http://mpp.cclearn.org/wp-content/uploads/2008/08/nezmc_report_card_on_dental_mercuryfinal.pdf

[B287] Bengtsson U The symbiosis between the dental and industrial communities and their scientific journals http://www.gbg.bonet.se/bwf/art/symbiosis.html

[B288] Consumer for Dental Choice Complaint against FDA 2008 http://www.toxicteeth.org/natcamp_fedgovt_fda_complaint_Dec07.cfm

[B289] FDI World Dental Federation FDI participates at WHO/UNEP meeting on future use of materials for dental restoration 2009 http://www.fdiworldental.org/content/fdi-participates-whounep-meeting-future-use-materials-dental-restoration

[B290] Mercury Policy Project Bender M Letter to WHO: WHO meeting report on the future of dental restorative materials 2010 http://mercurypolicy.org/wp-content/uploads/2010/12/letter_to_who_amalgam_nov_2010_final_final.pdf

[B291] Gruning T Gilmore AB McKee M Tobacco Industry Influence on Science and Scientists in Germany Am J Public Health 2006 96 20 32 10.2105/AJPH.2004.061507 16317203 PMC1470431

[B292] Hardell L Walker MJ Walhjalt B Friedman LS Richter ED Secret ties to industry and conflicting interests in cancer research Am J Ind Med 2007 50 227 233 10.1002/ajim.20357 17086516

[B293] Bohm SR Dian Z Gilman DS Maximizing profit and endangering health: corporate strategies to avoid litigation and regulation Int J Occup Environ Health 2005 11 338 348 16350467 10.1179/oeh.2005.11.4.338

[B294] Jacobson MF Lifting the veil of secrecy from industry funding of nonprofit health organizations Int J Occup Environ Health 2005 11 349 55 16350468 10.1179/oeh.2005.11.4.349

[B295] Egilman DS Bohme SR Over a barrel: corporate corruption of science and its effects on workers and the environment Int J Occup Environ Health 2005 11 331 337 16350466 10.1179/oeh.2005.11.4.331

